# Mitogenomic Insights into Phylogeny, Biogeography and Adaptive Evolution of the Genus *Typhlomys* (Rodentia: Platacanthomyidae)

**DOI:** 10.3390/ani15192823

**Published:** 2025-09-27

**Authors:** Chao Na, Xiaohan Wang, Yaxin Cheng, Yixin Huang, Shuiwang He, Laxman Khanal, Shunde Chen, Xuelong Jiang, Zhongzheng Chen

**Affiliations:** 1 Collaborative Innovation Center of Recovery and Reconstruction of Degraded Ecosystem in Wanjiang Basin Co-Founded by Anhui Province and Ministry of Education, School of Ecology and Environment, Anhui Normal University, Wuhu 241002, China; n1525620041@163.com (C.N.); wx374989939@163.com (X.W.); yaxin0701@ahnu.edu.cn (Y.C.); huangyx@ahnu.edu.cn (Y.H.); 2 State Key Laboratory of Genetic Resources and Evolution, Yunnan Key Laboratory of Biodiversity and Ecological Conservation of Gaoligong Mountain, Kunming Institute of Zoology, Chinese Academy of Sciences, Kunming 650204, China; heshuiwang@mail.kiz.ac.cn; 3 Central Department of Zoology, Institute of Science and Technology, Tribhuvan University, Kathmandu 44618, Nepal; khanal@ccdz.tu.edu.np; 4 International Centre for Biodiversity and Primates Conservation, Dali University, Dali 671003, China; 5 College of Life Sciences, Sichuan Normal University, Chengdu 610101, China; csd111@126.com

**Keywords:** ancestral area reconstruction, comparative mitogenomics, *ND5*, positive selection, *Typhlomys huangshanensis*

## Abstract

Soft-furred tree mice (genus *Typhlomys*), which are native to southern China and northern Vietnam, are unique rodents capable of echolocation. Little is known about their taxonomy, ecology, and natural history. In this study, we generated the complete mitochondrial genomes of seven species/putative species of *Typhlomys*. We conducted a comprehensive comparative analysis of these mitochondrial genomes focusing on sequence length, A+T content, A/T bias, A+T-rich regions, overlapping and intergenic spacer regions, nucleotide composition, relative synonymous codon usage, ancestral distributions, and the non-synonymous/synonymous substitution ratio (Ka/Ks). Additionally, we analyzed the phylogeny and adaptive evolution of these species/putative species. The mitogenomes of *Typhlomys* ranged from 16,487 to 17,380 bp in length, encoding the complete set of 37 genes typically found in mammalian mitogenomes. The base composition exhibited an A+T bias. The most frequently used codons were CUA (Leu), AGC (Ser), GGA (Gly) and UUA (Leu), UUG, CUG, CGU and GCG were the less frequently used codons. Ancestral distribution reconstruction suggests that *Typhlomys* originated in Central or Southwestern China. Notably, we found that the Ka/Ks ratio of the *ND5* gene in *T. huangshanensis* was greater than 1, indicating that this gene has undergone positive selection for efficient respiration in higher elevations and colder climates.

## Simple Summary

The genus *Typhlomys* includes small rodents from southern China and northern Vietnam known for their echolocation abilities. However, little is known about their taxonomy, life history, and environmental adaptations. This study analyzed the mitochondrial genomes of seven *Typhlomys* species to explore their phylogenetic relationships, origin, and adaptive traits. Results show stable genetic structures with variations in genome size and two distinct clades, including a potential new species. The genus likely originated in Central or southwestern China. Additionally, the ND5 gene in *Typhlomys huangshanensis* shows signs of positive selection, indicating adaptation to colder environments of higher elevation in the northernmost distribution limit of the genus. These findings enhance our understanding of *Typhlomys* and support conservation efforts.

## 1. Introduction

The soft-furred tree mice of the genus *Typhlomys* are a relict group within the family Platacanthomyidae [1]. This genus is notable among rodents, as it is currently the only known rodent taxon capable of echolocation [2,3]. *Typhlomys* is characterized by its small eyes and a distinctive terminal brush at the end of its tail [4]. These mice are known for their arboreal and burrowing lifestyle [5,6,7], and are mainly distributed in high mountain forests of southwestern China and northwestern Vietnam [4,8,9]. Among the seven species examined in this study namely: *T. chapensis*, *T. nanus*, and a putative species (*T.* sp. 2) inhabit temperate montane mixed forests with a humid climate, with habitats dependent on high-altitude bamboo groves, cave systems, and hollow trees. *T. fengjiensis* and *T. daloushanensis* occur in humid montane climates, which are characterized by hot-humid summers, mild winters, and high annual precipitation [9]. *T. cinereus* resides in ecotones between subtropical evergreen broad-leaved forests and bamboo groves, relying on thick leaf litter layers, rock crevices, and tree hollows, and its habitat is characterized by consistently high humidity [10]. *T. huangshanensis* inhabits mixed forests of Chinese red pines (*Pinus hwangshanensis*) and bamboo groves, showing high dependence on rock crevices and tree hollows; its habitat is characterized by year-round cloud cover and high humidity. Speciation among distinct species of this genus may be attributed to factors such as complex topography and climate. The type species of the genus, *Typhlomys cinereus*, was described by Milne-Edwards (1877) [11] based on the specimens collected from Guadun in Fujian Province in south China. *Typhlomys* was considered a monotypic genus for a long time, but in recent years there have been remarkable changes in its taxonomic understanding [4,7,9]. Currently, the genus contains seven established species: *T. chapensis*, *T. cinereus*, *T. daloushanensis*, *T. nanus*, *T. huangshanensis*, *T. fengjiensis*, *T. taxuansis*, and one putative species from Mt. Laojun and Dawei in Yunnan Province (*Typhlomys* sp. 2 hereafter) [4,7,9,12]. To date, studies on *Typhlomys* are mainly focused on taxonomy; however, the phylogenetic relationships of some species are still unresolved [7,9] and little is known about their ecology and natural history.

Mitochondrial genomes are widely used in the study of population genetic structure and the reconstruction of phylogenetic relationships because of their simple structure, short length, fast evolutionary rate and matrilineal inheritance. One of the functions of mitochondria is to supply energy by serving as an important site for the oxidative phosphorylation system (OXPHOS), which makes them highly sensitive to energy-related selective pressures. Approximately 95% of the energy demand for animals to engage in locomotion is produced via the mitochondrial respiratory chain. The 13 PCGs (protein-coding genes) are closely related to the process of OXPHOS, and thus these subunits are constantly under positive or negative selective pressure [13]. Many previous studies have detected positive selection signals in mitochondrial genes [12,14,15,16], indicating that positive selection plays a strong role in species adaptation. However, studies on the mitochondrial genome of *Typhlomys* are scarce, only one incomplete mitogenome of *Typhlomys cinereus* is available in NCBI GenBank [17] (GenBank accession no. KX397283).

In the current study, we performed a comparative mitogenomic analysis on the genus *Typhlomys* to (1) characterize the features and distinct nucleotide patterns in their mitochondrial genomes; (2) reconstruct the phylogeny based on mitochondrial genes and estimate the divergence time and the biogeographical history; and (3) detect whether there is any adaptive selection of the mitochondrial genes.

## 2. Materials and Methods

### 2.1. Sampling and DNA Extraction

**Figure 1:**
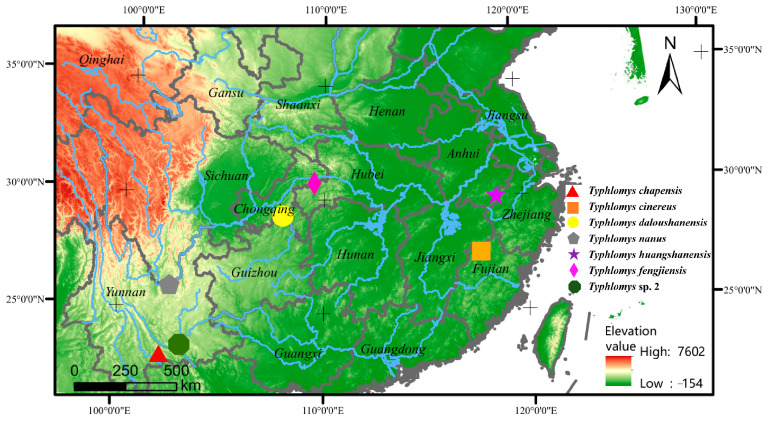
Sample localities of *Typhlomys* in this study.

Seven specimens representing six recognized species and one putative species of *Typhlomys* were collected from Yunnan, Anhui, Fujian, and Chongqing in China (Figure 1, Table 1). Muscle tissues were sampled and stored in 99.99% ethanol for molecular studies. Total genomic DNA was extracted from the preserved samples using the Qiagen DNeasy Blood and Tissue Kits (Qiagen China, Shanghai, China).

### 2.2. Sequencing and Assembly

After quantification of the extracted DNA, we employed two strategies to obtain the mitochondrial genome of *Typhlomys* species. Four of them (*T. daloushanensis*, *T. cinereus*, *T. nanus*, *T.* sp. 2) were obtained using a long-range PCR and cross-species hybridization capture following the method described by Chen et al. [18], and the other two (*T. chapensis* and *T. huangshanensis*) were obtained using the next-generation sequencing (NGS) on an Illumina Novaseq platform with a strategy of 150bp paired-ends [19]. The assembly of the mitochondrial genome was performed using Novoplasty 4.3.1 [20].

### 2.3. Mitochondrial Genome Annotation

Twenty-two tRNA genes were identified with the use of MITOS WebServer (http://mitos.bioinf.uni-leipzig.de, accessed on 29 November 2023), setting the parameters with the Vertebrate Mito genetic code [21]. Their secondary structures were plotted manually from the MITOS predictions using Adobe Illustrator. Every sequence of tRNA genes was manually checked separately. Protein-coding genes (PCGs) were identified as open reading frames corresponding to the 13 PCGs in the metazoan mitochondrial genome. The rRNA genes and control region were identified by the boundaries of the tRNA genes.

### 2.4. Comparative Analysis

Base composition and relative synonymous codon usage (RSCU) were calculated using MEGA 11 [22]. The relative composition of different bases was measured in terms of GC and AT skews according to the formulae suggested by Hassanin et al. [23]: GC-skew = (G − C)/(G + C) and AT-skew = (A − T)/(A + T). The number of synonymous substitutions per synonymous site (Ks) and the non-synonymous substitutions per non-synonymous site (Ka) for each of the concatenated 13 PCGs of the *Typhlomys* mitogenomes were calculated by DnaSP v.6.12.03 [24].

### 2.5. Phylogenetic Analysis

We performed maximum likelihood (ML) and Bayesian inference (BI) analyses to reconstruct the phylogenetic relationships of *Typhlomys*. The mitogenomes of *Rattus rattus*, *Myosplax aspalax* and *Sicista concolor* were downloaded from GenBank and used as outgroups. The ML and BI analyses were conducted on the concatenated dataset of 13 PCGs and two rRNA using IQ-TREE [25] and MrBayes [26], respectively, in PhyloSuite [27]. The best-fit partitioning scheme and evolutionary substitution models were estimated using PartitionFinder 2 [28] under the Bayesian Information Criterion (BIC, Supplementary Table S13). FigTree v. 1.4.4 [29] was used to visualize the resulting trees.

### 2.6. Divergence Time Estimation

We estimated divergence times of *Typhlomys* based on the concatenated dataset of 13 PCGs and two rRNA in BEAST 2.6 [30]. We defined data blocks based on genes and codon positions, and the best-fit partitioning scheme and substitution models using PartitionFinder 2. Divergence dates were calibrated based on two secondary calibration points: (1) the split of the most recent common ancestor Myomorpha is estimated at about 45.0 Ma [31], we established the prior using an exponential distribution prior (mean = 4.64, offset = 45.0) following Cheng et al. [4], and (2) the most recent common ancestor of *Typhlomys* is estimated at about 12.6 Ma ((95% CI = 8.9–16.09) [4]; we established the prior using a lognormal distribution (mean:12.5, standard deviation: 0.19, offset: 0), such that the median age was at 12.5 Ma and the 95% Cl was 8.98–16.8 Ma. We repeated the analysis twice; each analysis consisted of 100 million generations and sampled every 10,000 generations. The first 10% of the generations were discarded as burn-in. Convergence was assessed using Tracer v1.7.2 [32] and effective sample size values greater than 200 were considered adequate.

### 2.7. Ancestral Distribution Analysis

To investigate the biogeographical history of *Typhlomys*, we reconstructed the ancestral distribution of *Typhlomys* using BioGeoBEARS (implemented in RASP 4.3) [33]. The DIVALIKE model was used in our analysis due to its highest AICc_wt among the six candidate models (i.e., DEC, DEC+j, DIVALIKE, DIVALIKE+J, BAYAREALIKE, and BAYAREALIKE+J) [34,35,36]. Four geographical regions were determined in the analysis of ancestral distributions: (A) Central China; (B) Southwestern China and Northern Vietnam; (C) Eastern China; (D) Southern China. The coalescent-based Bayesian phylogenetic tree was used as the base topology. The other settings applied the default values of the software.

### 2.8. Selection Pressure Analysis

We used the ratio of non-synonymous/synonymous substitution ratio (Ka/Ks) to estimate sequence evolution following Wang et al. [37]. Values of Ka/Ks > 1 imply the presence of positive selection; Ka/Ks = 1 imply neutrality; and Ka/Ks < 1 indicating the presence of purifying selection [38]. To ensure an accurate and unbiased comparison, we used *Rattus rattus*, *Myosplax aspalax* and *Sicista concolor* as reference sequences to calculate Ka/Ks ratios for each PCG. For the selection analysis of the 13 PCGs, the stop codon was removed, and the NADH dehydrogenase 6 (*ND6*) sequence was reverse complemented because *ND6* is on the minority-strand (N-strand). The overlapping regions (43 bp of *ATP8*/*ATP6* and 7 bp of *ND4L*/*ND4*) were included twice in the comparison to enable analysis of all codons.

## 3. Results

### 3.1. Genome Structure and Organization

**Figure 2:**
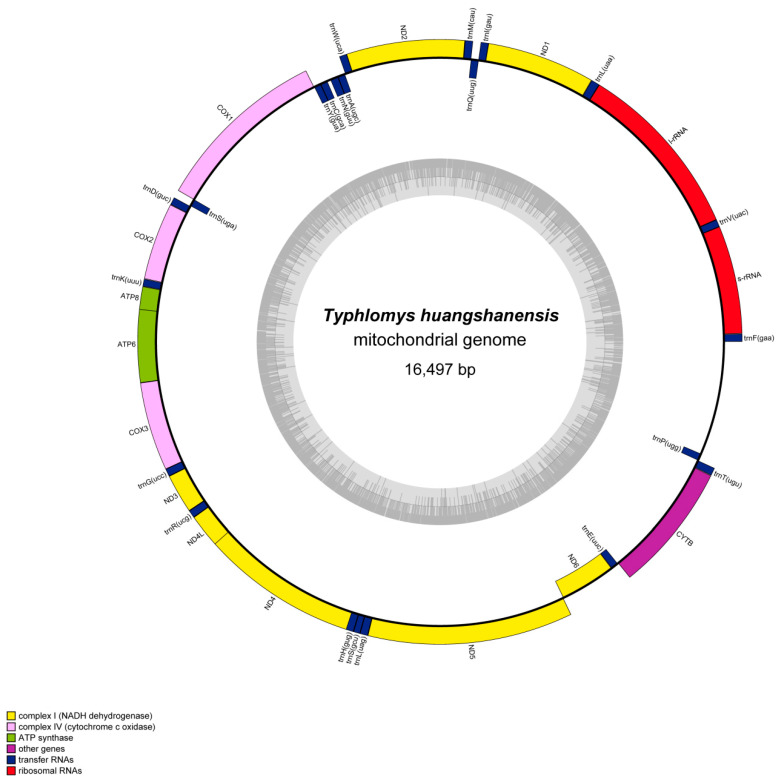
Circular maps of the mitochondrial genome *T. huangshanensis* (QLF1911102). Protein-coding and ribosomal genes are indicated using standard abbreviations. The J-strand is shown on the outer circle and the N-strand on the inner circle.

Mitogenome lengths of *Typhlomys* ranged from 16,490 bp in *T.* sp. 2 to 17,380 bp in *T. chapensis* (Table 2). All of them contained the complete set of 37 genes common to mammalian mitogenomes, including 13 protein-coding genes (PCGs), 22 transfer RNA (tRNA) genes and two ribosomal RNA (rRNA) genes. There are 28 genes (12 PCGs, 14 tRNAs and 2 rRNAs) encoded by the H-strand, and 9 genes (1 PCG and 8 tRNAs) encoded by the L-strand, making the entire genome a typical double-stranded circular molecular structure (Figure 2, Supplementary Figures S1–S6). The gene arrangement, organization, and content within the mitogenomes were in line with those of other rodent mitogenomes.

The nucleotide composition of all the mitogenomes had a high A+T content, with an average of 63.1%, showing a strong A/T bias (Table 2). Among the mitogenomes, *T. cinereus* had the highest A+T content (64.6%) whereas *T. chapensis* had the lowest (60.6%). For the whole mitochondrial genome, all AT-skews were positive, ranging from 6.36% (*T. huangshanensis*) to 10.4% (*T. chapensis*) and while all GC-skews were negative, ranging from −36.4% (*T. daloushanensis*) to −34.7% (*T. huangshanensis*), indicating that the mitogenomes of *Typhlomys* were biased toward A and T.

### 3.2. Protein-Coding Genes (PCGs)

The 13 PCGs of *Typhlomys* mitogenomes contain three cytochrome c oxidase subunits, seven NADH dehydrogenase subunits, two ATPase subunits and one cytochrome b gene, which is similar to other mammals in Rodentia. Most PCGs start with ATG and end with TAA (Table 3, Supplementary Tables S7–S12). While a few PCGs in the mitogenomes use ATT, ATA, CCT, and GTG as the start codon, and TAG, AGG, TTA, and CAT as termination codon.

**Figure 3:**
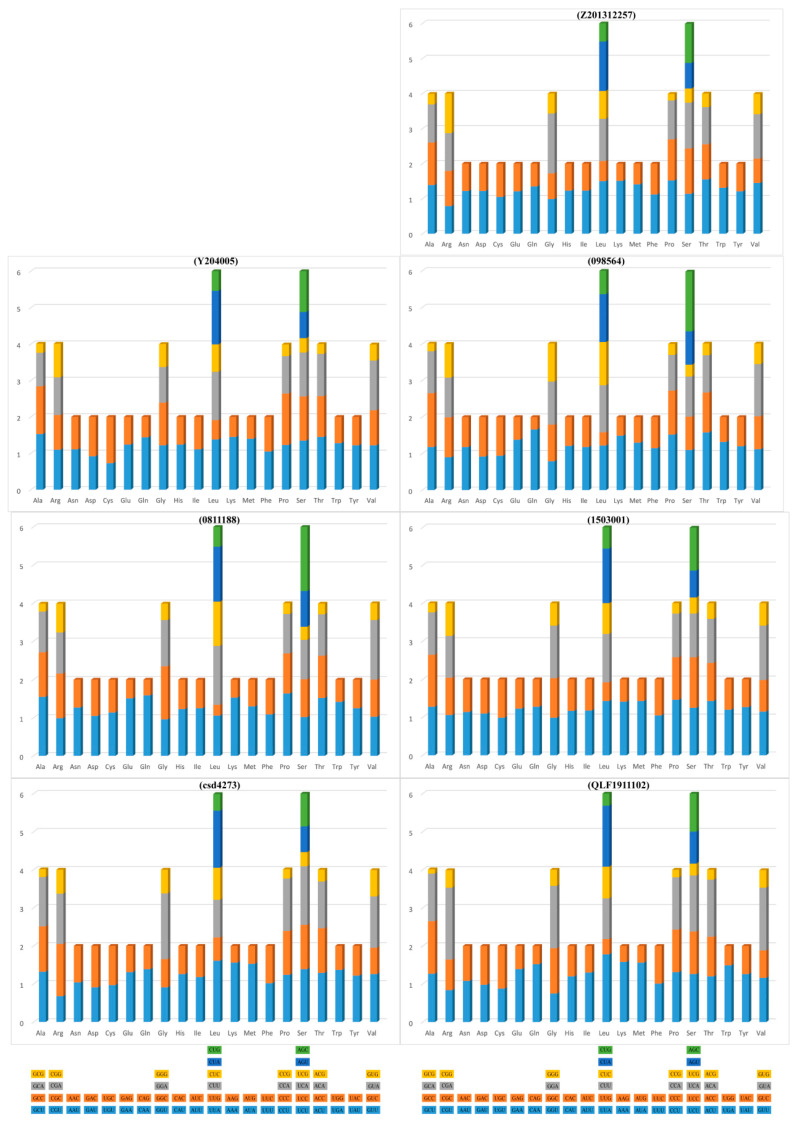
Relative synonymous codon usage (RSCU) of the mitochondrial genomes of *T. chapensis* (098564), *T. cinereus* (Z201312257), *T. daloushanensis* (Y204005), *T. nanus* (0811188), *T. huangshanensis* (QLF1911102), *T. fengjiensis* (csd4273), and *T.* sp. 2 (1503001).

The average A+T content of PCGs in *Typhlomys* is 62.4%. To further investigate this high A and T content and the frequency of synonymous codon usage, we calculated the relative synonymous codon usage (RSCU) values. The relative synonymous codon usage (RSCU) of *Typhlomys* is shown in Figure 3. Overall, the most frequently used codons were CUA (Leu), AGC (Ser), GGA (Gly) and UUA (Leu), whereas codons ending in G or C, UUG, CUG, CGU and GCG were the less frequently used codons. The predominance of codons ending in A or T leads, at least in part, to a preference for A and T.

### 3.3. Ribosomal RNA and Transfer RNA

**Figure 4:**
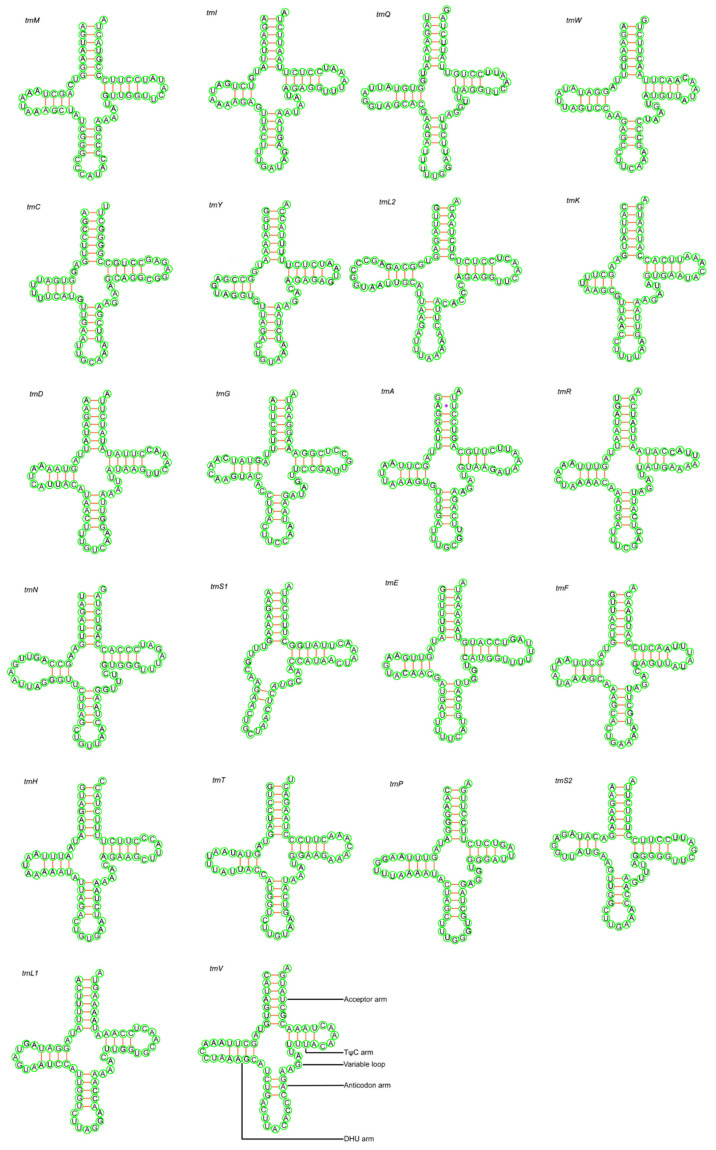
Predicted secondary cloverleaf structure for the tRNAs of *T. huangshanensis* (QLF1911102).

The full length of rRNA genes was between 2525 and 2540 bp and consist of two subunits, 12S rRNA (955–966 bp) and 16S rRNA (1570–1575 bp). The nucleotide composition of 12S rRNA and 16S rRNA is skewed with respect to the A+T content. The AT-skew of 12S rRNA and 16S rRNA were positive, while the GC-skew was negative, suggesting that adenine and cytosine are relatively more prevalent in rRNA than thymine and guanine (Table 4, Supplementary Tables S1–S6). There were 22 tRNA genes in the mitochondrial genome, most of which were encoded by the H-strand (trnF, trnV, trnL 2, trnI, trnM, trnW, trnD, trnK, trnG, trnR, trnH, trnS 1, trnL 1, trnT), while the trnQ, trnA, trnN, trnC, trnY, trnS 2, trnE, and trnP were encoded by the L-strand. All tRNA genes exhibit a cloverleaf secondary structure, except for trnS 1, which lack a stabilizing dihydrouridine arm loop (Figure 4, Supplementary Figures S7–S12). For tRNAs, the AT skewness is positive, and the GC skewness is negative, again indicating that adenine and cytosine are relatively more prevalent in rRNAs than thymine and guanine.

### 3.4. Overlapping and Intergenic Spacer Regions

We observed a total of 105 intergenic spacer, ranging in size from 1 to 335 bp, in the mitochondrial genomes of *Typhlomys*. The longest intergenic spacer (335 bp) was observed in *T. daloushanensis*, between the trnP and trnF genes (Table 3, Supplementary Tables S7–S12).

In all species of *Typhlomys*, there were 69 overlapping gene regions with lengths ranging from 1 to 43 bp. The longest overlapping sequence in each genome was between ATP8 and ATP6, 43 bp. The number of gene overlaps ranged from 9 to 10 among different species, and the total length was 61–74 bp.

### 3.5. A+T Rich Region

The A+T-rich region, also known as the control region, is positioned between the tRNAs trnP and trnF. The lengths of the control region ranged between 625 bp in *T. fengjiensis* and 1064 bp in *T. huangshanensis* (Table 4, Supplementary Tables S1–S6). The A+T content of control region of the seven species ranged from 62.2% (*T. chapensis*) to 69.5% (*T. huangshanensis*).

### 3.6. Phylogenetic Analyses

**Figure 5:**
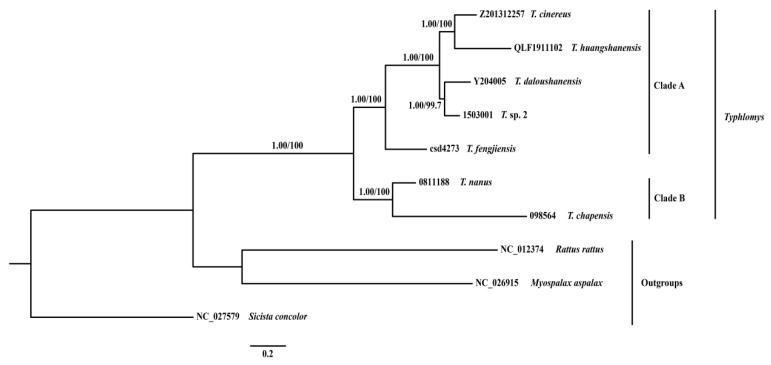
Phylogenetic tree produced by maximum likelihood and Bayesian inference analyses based on 13 PCGs and two rRNA. Posterior probability (PP) values and bootstrap (BS) are shown on the nodes.

We considered posterior probabilities (PP) ≥ 0.95, SH-like approximate likelihood-ratio test values (SH-aLRT) ≥ 80 and ultrafast bootstrap supports (UFBoot) ≥ 95 as strong support [36]. The Bayesian inference and maximum likelihood phylogenetic trees showed similar topologies (Figure 5). All seven species/putative species of *Typhlomys* were well supported in the phylogenetic trees (PP > 0.99, UFBoot = 10,000, SH-aLRT = 1000). The seven species under the genus *Typhlomys* were divided into two clades. One clade comprises *T. cinereus*, *T. huangshanensis*, *T. daloushanensis*, *T. fengjiensis* and *T.* sp. 2 (Clade A), and the other comprises *T. chapensis* and *T. nanus* (Clade B). *Typhlomys fengjiensis* was revealed in a basal position of Clade A and was sister to the rest species in the clade (PP > 0.99, UFBoot = 10,000, SH-aLRT = 1000).

**Figure 6:**
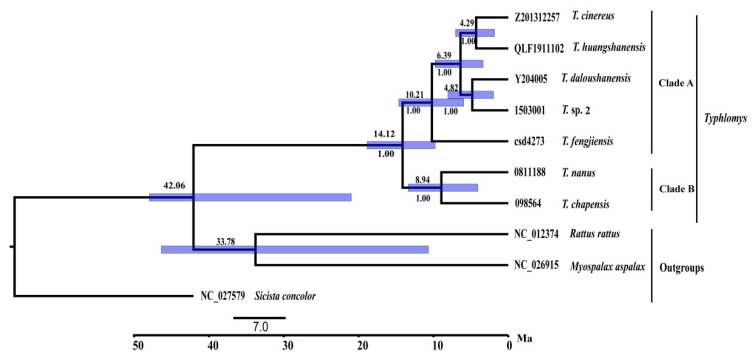
Divergence times estimated using BEAST based on 13 PCGs and two rRNA sequences. Branch lengths represent time. Numbers above the nodes indicate median divergence time. Numbers below the nodes represent the posterior probabilities (PP).

The divergence time analyses showed divergence within *Typhlomys* started during the middle Miocene at about 14.12 Ma (95% CI = 9.77–18.87 Ma), with the remaining species diverging between 4.29 and 10.21 Ma (95% CI = 1.84–14.65 Ma) (Figure 6).

### 3.7. Ancestral Distributions

**Figure 7:**
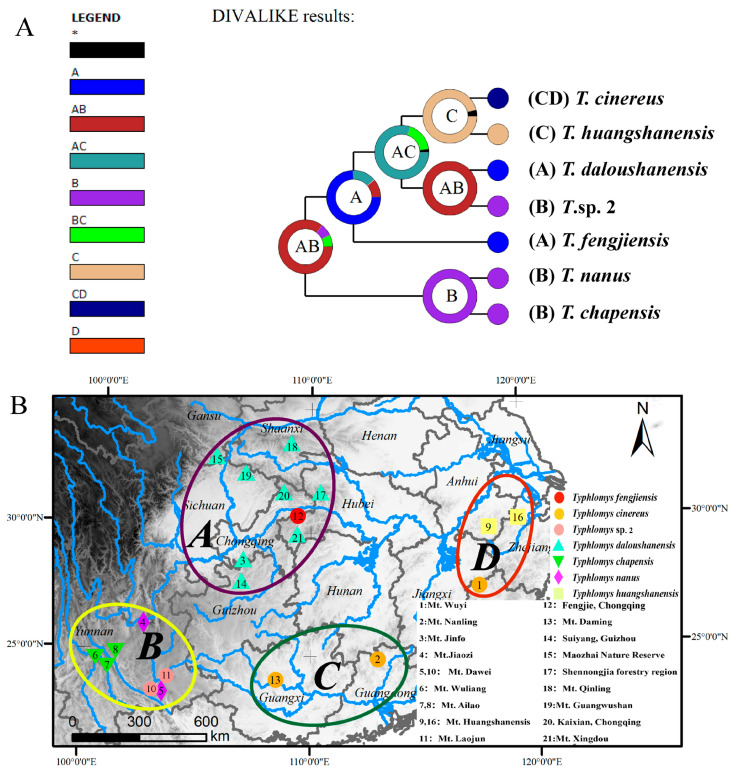
Reconstruction of the ancestral distribution of *Typhlomys*. (**A**) geographical evolutionary history inferred based on DIVALIKE. (**B**) Ancestral distribution range based on the Bayesian binary MCMC method. The distribution range is based on the majority tree annotated from Bayesian phylogenetic analysis. The classifications of the geographical regions of current samples are identified in different colors: (A) *T. daloushanensis* and *T. fengjiensis* of Central China; (B) *T. chapensis*, *T. nanus* and *T.* sp. 2 of Southwestern China; (C) *T. cinereus* and *T. huangshanensis* of Southern China; (D) *T. cinereus* of Eastern China. Black with an asterisk * represents other ancestral ranges.

Based on AICc model comparisons, the DIVALIKE model demonstrated an optimal fit to our dataset under the “BioGeoBEARS” framework (AICc_wt = 0.42; Supplementary Table S14). Results revealed that the ancestral distribution of *Typhlomys* most likely originated in the Central and Southwestern China (regions A and B) with a probability of 87.12% (Figure 7). Furthermore, the origin of *T. chapensis* and *T. nanus* most likely occurred in Southwestern China (region B), supported by 100% probability. The ancestor of the *T. cinereus*, *T. huangshanensis*, *T. daloushanensis*, *T. fengjiensis* and *T.* sp. 2 potentially occurred in the area around Central China (region A), with a probability of 77.47%.

### 3.8. Selection Pressure in Mitochondrial Genes

**Figure 8:**
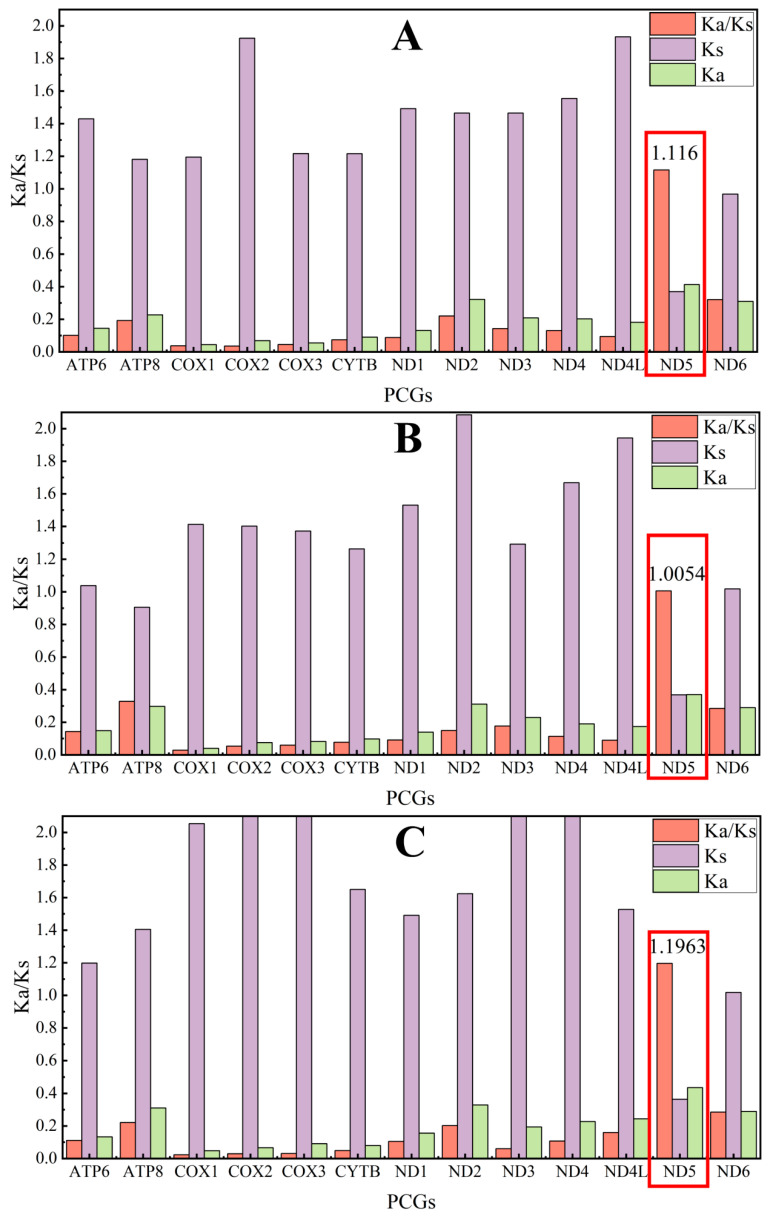
Evolutionary rates of 13 protein-coding genes in the mitochondrial genome of *T. huangshanensis* (QLF19111002). (**A**) *Rattus rattus* as outgroup; (**B**) *Myospalax aspalax* as outgroup; (**C**) *Sicista concolor* as outgroup. Genes with a ka/ks ratio greater than 1 have been marked with red rectangles.

Selection pressure analyses were conducted using *Rattus rattus*, *Myospalax aspalax*, and *Sicista concolor* as outgroups. All the results showed that Ka/Ks of ND5 > 1 in *T. huangshanensis* (Figure 8). It indicated that the *ND5* gene which codes for *ND5* protein, an enzyme complex in the mitochondria that is responsible for electron transport generating ATP was subjected to positive selection. The remaining 12 PCGs of *T. huangshanensis*, as well as the 13 PCGs of the other six *Typhlomys*, had the Ka/Ks < 1, indicating that they were subjected to purifying selection.

## 4. Discussion

In this study, we present a comparative analysis of the mitochondrial genomes of the species under the genus *Typhlomys*. We sequenced the mitogenomes of six recognized and one putative species of *Typhlomys* and analyzed their composition and evolutionary relationships. The gene arrangement, organization, and content of the seven mitochondrial genomes is like that of other mammals [39], illustrating the highly conserved nature of the mammalian mitochondrial genome. The size of the mitogenomes varied among the examined species, ranging from 16,487 bp in *T. fengjiensis* to 17,380 bp in *T. chapensis*. Compared with other species, the length of *T. chapensis* mitochondrial genome is longer by approximately 800 bp (Supplementary Table S15) mainly due to longer (1935 bp) D-loop region of the species. D-loop region is the largest non-coding region, located between trnP and trnF in these mitogenomes, which is supposed to contain regulatory elements related to the control of replication and transcription [40], and is frequently used in vertebrate phylogenetic and phylogeographic studies due to higher rates of mutation in comparison to the coding regions [41].

It is suggested that the variation in codon usage bias intensity among taxa is dictated by the variation in selective pressure [42,43]. The relative synonymous codon usage (RSCU) and codon distribution of the seven mitochondrial genomes showed that CUA (Leu), AGC (Ser), GGA (Gly) and UUA (Leu) are the most prevalently used codons, and on the other hand, codons UUG, CUG, CGU and GCG which terminate with G or C are less frequently utilized. Additionally, the codons exhibited a preference for A/T over G/C, thereby causing the A+T content to be greater than the G+C content in the PCGs. GC pairs are considered more stable than AT pairs, and thus high AT content of *Typhlomys* may produce more active structures that accumulate evolutionary adaptations [44].

Consistent with Pu et al. [9], our phylogenetic tree constructed on the concatenated dataset of 13 PCGs and two rRNA supported seven species of *Typhlomys* clustered into two major clades; one clade was formed by *T. cinereus*, *T. huangshanensis*, *T. daloushanensis*, *T. fengjiensis* and *T.* sp. 2 (Clade A); the other clade comprised *T. chapensis* and *T. nanus* (Clade B), the two are sister groups to each other. Pu et al. [9] revealed the *T. daloushanensis* is sister to *T. fengjiensis* using three mitochondrial genes (*Cyt b*, *COI*, and *ND2*), with low bootstrap supports (BS = 71). However, our results revealed *T. fengjiensis* is on the basal position of Clade A. Substantially, the putative species (*T.* sp. 2) from Mt. Dawei is strongly supported as sister to *T. daloushanensis* (BS = 99.7), compared with previous studies, this result is more reliable. This result suggests that it may represent a distinct species and that a detailed taxonomic study is required.

To investigate the role of selection pressure and the evolution of the mitochondrial genomes in the genus *Typhlomys*, we estimated the Ka/Ks values of each protein-coding gene (PCG) across seven species in the context of different outgroups. Our analysis revealed that only the Ka/Ks of the *ND5* in *T. huangshanensis* consistently exceeded 1 when compared with different outgroups, indicating that this gene was under positive selection [44]. The *ND5* gene, as an important component of the mitochondrial complex I proton channel [45], has adapted to the living environment that requires a large amount of ATP by increasing the amount of proton transport, and thus is reinforced by positive selection, which leads to a greater rate of non-synonymous than synonymous substitutions. We further conducted a comparative analysis of the mitochondrial gene structure of the *ND5* gene across seven species (Supplementary Table S16). The results revealed that *T. huangshanensis* exhibits the highest AT content and the lowest GC content in the *ND5* gene. This might indicate that the *ND5* gene of *T. huangshanensis* has been subjected to a stronger AT-biased mutation pressure during evolution because AT base pairs, which contain only two hydrogen bonds, are more prone to breakage than GC base pairs (with three hydrogen bonds), leading to mismatches during DNA replication. Higher AT content might facilitate accelerating the transcription process and enhancing the protein synthesis rate, thereby enabling the rapid replenishment of energy consumption [46].

*T. huangshanensis* has so far been recorded only in the Huangshan Mountains (Anhui Province) and Qingliang Peak (Zhejiang Province), making it the northernmost-distributed species within the genus *Typhlomys*. Its habitat consists of mixed forests dominated by Chinese red pines with interspersed bamboo groves; this vegetation structure provides *T. huangshanensis* with diverse food resources and shelter sites. Among the seven species investigated in this study, *T. huangshanensis* exhibits a middle-range altitudinal distribution. Notably, it has the northernmost distribution compared to other congeneric species, which necessitates its adaptation to multiple stressors including hypoxia and low temperatures. Therefore, the positive selection observed in the *ND5* gene may be associated with the enhanced efficiency of energy metabolism in this species under such complex environmental conditions. Meanwhile, *T. huangshanensis* may encounter unique competitive or predatory pressures within its ecological niche. It is thus hypothesized that the positive selection of the ND5 gene is linked to the species’ specific activity patterns and survival strategies.

Based on the results of ancestral distribution analysis, *T. huangshanensis* is highly likely to have originated from the dispersal of species from central or southwestern China to the eastern region. This species distributed solely in eastern China is also a peripheral species representing the northernmost distribution record of the genus *Typhlomys*. During the long-term dispersal process, the complex topography of mountain ranges and changes in climatic conditions may have promoted allopatric speciation, ultimately leading to the emergence of narrowly distributed endemic species. We hypothesize that in this “new” environment, *T. huangshanensis* has adapted to the ecological conditions, and its *ND5* gene has been subjected to positive selection pressure. Further research is still needed in the future to clarify the natural adaptation mechanisms of *T. huangshanensis*.

## 5. Conclusions

This study generated complete mitochondrial genomes of seven *Typhlomys* species/putative species, filling gaps in the genus’ mitogenomic data. All mitogenomes (16,487–17,380 bp) have the typical mammalian 37-gene structure, with size variation driven by the A+T-rich control region and strong A+T bias average 63.1% reflected in frequent CUA, AGC, GGA, UUA codons. Phylogenetic analyses (ML/BI) resolve *Typhlomys* into two clades, confirming *T.* sp. 2’s distinctness, and diversification began 14.12 Ma (middle Miocene), matching southern China’s tectonic/climatic changes. Ancestral reconstruction (DIVALIKE model) points to a Central/Southwestern China origin (87.12% probability) with later range expansions, while selection analyses identify positive selection on *T. huangshanensis*’ *ND5* (Ka/Ks > 1) to enhance energy metabolism for high-altitude, cold habitats in the northernmost latitude of the distribution range of the genus. These findings clarify *Typhlomys’* phylogeny, biogeography, and adaptive evolution, supporting future ecological and conservation studies.

## Figures and Tables

**Table 1:** Samples and sequences used in this study. All sequences of *Typhlomys* were newly sequenced and have been submitted to the NCBI GenBank with following accession numbers.

Species	Field Number	GenBank Accession No.	Location	Latitude (N)	Longitude (E)	Elevation
*Typhlomys chapensis*	098564	PQ825594	Mt. Huanglian, Yunnan	22.87	103.24	2232
*Typhlomys cinereus*	Z201312257	PQ825595	Mt. Wuyi, Fujian	27.64	117.90	400
*Typhlomys daloushanensis*	Y204005	PQ825596	Mt. Dalou, Chongqing	29.02	107.19	2105
*Typhlomys nanus*	0811188	PQ825597	Mt. Jiaozi, Yunnan	26.07	102.83	3204
*Typhlomys huangshanensis*	QLF1911102	PQ825598	Mt. Qingliangfeng, Anhui	30.13	118.85	1303
*Typhlomys fengjiensis*	CSD4273	PQ825599	Fenjie, Chongqing, China	30.66	109.52	1883
*Typhlomys* sp. 2	1503001	PQ825600	Mt. Dawei, Yunnan	23.30	103.95	2043
*Rattus rattus*		NC_012374				
*Myosplax aspalax*		NC_026915				
*Sicista concolor*		NC_027579				

**Table 2:** Nucleotide composition of seven generated mitogenomes.

Taxa	Length (bp)	A%	C%	G%	T%	G+C%	A+T%
*T. chapensis*	17,380	33.5	26.8	12.6	27.2	39.4	60.6
*T. cinereus*	16,776	34.7	24.1	11.3	29.9	35.4	64.6
*T. daloushanensis*	16,984	34.2	25.3	11.8	28.7	37.1	62.9
*T. nanus*	16,665	33.9	25.9	12.3	28.0	38.2	61.8
*T. huangshanensis*	16,497	34.3	23.9	11.6	30.2	35.5	64.5
*T.* *fengjiensis*	16,487	34.5	24.5	11.7	29.3	36.2	63.8
*T.* sp. 2	16,490	34.3	24.7	11.8	29.9	36.5	63.5

**Table 3:** Mitogenomic organization of *T. huangshanensis* (QLF1911102).

	Position		Strand	Length	Intergenic Nucleotides	Codons	
	From	To				Start	Stop
trnF	1	69	+	69	2		
rrnS	72	1026	+	955	−1		
trnV	1026	1092	+	67	0		
rrnL	1093	2667	+	1575	0		
trnL2	2668	2742	+	75	1		
ND1	2744	3700	+	957	−2	GTG	TAG
trnI	3699	3767	+	69	−3		
trnQ	3765	3836	−	72	−1		
trnM	3836	3905	+	70	0		
ND2	3906	4949	+	1044	−2	ATT	TAG
trnW	4948	5015	+	68	5		
trnA	5021	5089	−	69	0		
trnN	5090	5163	−	74	2		
trnC	5196	5263	−	68	0		
trnY	5264	5327	−	64	1		
COX1	5329	6873	+	1545	−3	ATG	TAA
trnS2	6871	6939	−	69	3		
trnD	6943	7011	+	69	1		
COX2	7013	7696	+	684	2	ATG	TAA
trnK	7699	7763	+	65	1		
atp8	7765	7968	+	204	−43	ATG	TAA
atp6	7926	8606	+	681	−1	ATG	TAA
COX3	8606	9390	+	785	−1	ATG	TTA
trnG	9390	9457	+	68	0		
ND3	9458	9805	+	348	0	ATT	TAA
trnR	9806	9874	+	69	0		
ND4L	9875	10,171	+	297	−7	ATG	TAA
ND4	10,165	11,542	+	1383	0	ATG	ACT
trnH	11,543	11,610	+	68	0		
trnS1	11,611	11,669	+	59	0		
trnL1	11,670	11,739	+	70	0		
ND5	11,740	13,551	+	1812	1	ATT	TAA
ND6	13,553	14,077	−	525	1	TCT	CAT
trnE	14,079	14,148	−	70	5		
Cytb	14,154	15,293	+	1140	2	ATG	AGA
trnT	15,296	15,365	+	70	1		
trnP	15,367	15,433	−	67	321		

**Table 4:** Nucleotide composition on mitogenome of *T. huangshanensis* (QLF1911102).

Regions	Size (bp)	A (%)	C (%)	G (%)	T (%)	A+T (%)	C+G (%)	AT-Skew	GC-Skew
Mitochondrial genome	16,497	34.3	23.9	11.6	30.2	64.5	35.5	6.36	−34.7
PCGs	11,405	32.8	25.8	10.5	31.0	63.8	36.2	2.82	−42.3
PCGs(J)	10,880	32.4	25.6	10.6	31.3	63.8	36.2	1.72	−41.4
PCGs(N)	525	40.8	29.7	6.7	22.9	63.6	36.4	28.1	−63.2
tRNAs	1509	36.4	20.1	13.9	29.6	66.0	34.0	10.3	−18.2
tRNAs(J)	956	37.4	17.4	14.4	30.8	68.2	31.8	9.68	−9.43
tRNAs(N)	553	34.7	24.8	13.0	27.5	62.2	37.8	11.6	−31.2
rRNAs	2530	37.9	19.4	16.0	26.7	64.6	35.4	17.3	−9.6
A+T	1064	38.3	20.8	9.7	31.3	69.5	30.5	/	/

## Data Availability

All the New DNA sequences (accession numbers: PQ825594-PQ825594600) used in this study were deposited in GenBank (https://www.ncbi.nlm.nih.gov/, accessed on 3 January 2025).

## Supplementary Materials

The following supporting information can be downloaded at https://www.mdpi.com/article/10.3390/ani15192823/s1, Figure S1: Circular maps of the mitochondrial genome *T. chapensis* (098564); Figure S2: Circular maps of the mitochondrial genome *T. daloushanensis* (Y204005); Figure S3: Circular maps of the mitochondrial genome *T. nanus* (0811188). Figure S4: Circular maps of the mitochondrial genome *T. cinereus* (Z201312257); Figure S5: Circular maps of the mitochondrial genome *T. fengjiensis* (CSD4273); Figure S6: Circular maps of the mitochondrial genome *T.* sp. 2 (1503001); Figure S7: Predicted secondary cloverleaf structure for the tRNAs of *T. cinereus* (Z201312257); Figure S8: Predicted secondary cloverleaf structure for the tRNAs of *T. daloushanensis* (Y204005); Figure S9: Predicted secondary cloverleaf structure for the tRNAs of *T. chapensis* (098564); Figure S10: Predicted secondary cloverleaf structure for the tRNAs of *T. nanus* (0811188); Figure S11: Predicted secondary cloverleaf structure for the tRNAs of *T.* sp. 2 (1503001); Figure S12: Predicted secondary cloverleaf structure for the tRNAs of *T. fengjiensis* (csd4273); Table S1: Nucleotide composition of separate regions on mitogenome of *T. daloushanensis* (Y204005); Table S2: Nucleotide composition of separate regions on mitogenome of *T. chapensis* (098564); Table S3: Nucleotide composition of separate regions on mitogenome of *T. nanus* (0811188); Table S4: Nucleotide composition of separate regions on mitogenome of *T. fengjiensis* (csd4273); Table S5: Nucleotide composition of separate regions on mitogenome of *T. cinereus* (Z201312257); Table S6: Nucleotide composition of separate regions on mitogenome of *T.* sp. 2 (1503001); Table S7: Mitogenomic organization of *T. daloushanensis* (Y204005); Table S8: Mitogenomic organization of *T. chapensis* (098564); Table S9: Mitogenomic organization of *T. nanus* (0811188); Table S10: Mitogenomic organization of *T. fengjiensis* (csd4273); Table S11: Mitogenomic organization of *T. cinereus* (Z201312257); Table S12: Mitogenomic organization of *T.* sp. 2 (1503001); Table S13: Partitioning schemes and evolutionary models for Mitochondrial; Table S14: Comparison of the fit of different models of biogeographical range evolution and model specific estimates for different parameters (d = dispersal, e = extinction, j = weight of jump dispersal (founder speciation)); Table S15: Comparison of the length of the D-loop region; Table S16: Compare the *ND5* in the mitochondrial genomes of various species.

## References

[1] Musser G.G., Carleton M.D., Wilson D.E., Reeder D.M. (2005). Superfamily Muroidea. Mammal Species of the World: A Taxonomic and Geographic Reference.

[2] Panyutina A.A., Kuznetsov A.N., Volodin I.A., Abramov A.V., Soldatova I.B. (2017). A blind climber: The first evidence of ultrasonic echolocation in arboreal mammals. Integr. Zool..

[3] He K., Liu Q., Xu D.M., Qi F.Y., Bai J., He S.W., Chen P., Zhou X., Cai W.Z., Chen Z.Z. (2021). Echolocation in soft-furred tree mice. Science.

[4] Cheng F., He K., Chen Z.Z., Zhang B., Wan T., Li J.T., Zhang B.W., Jiang X.L. (2017). Phylogeny and systematic revision of the genus *Typhlomys* (Rodentia, Platacanthomyidae), with description of a new species. J. Mammal..

[5] Smith A.T., Xie Y. (2013). A Guide to the Mammals of China.

[6] Abramov A., Balakirev A., Rozhnov V. (2014). An enigmatic pygmy dormouse: Molecular and morphological evidence for the species taxonomic status of *Typhlomys chapensis* (Rodentia: Platacanthomyidae). Zool. Stud..

[7] Balakirev A.E., Phuong B.X., Rozhnov V.V. (2024). *Typhlomys* (Rodentia, Platacanthomyidae): New species of the genus from northern Vietnam with notes on conservation status and distribution. Biodivers. Data. J..

[8] Su W.T., Chen Z.Z., Wan T., Wang X., Zhou H.Y., Hu Y., Wang J.H., Jiang X.L., Nie W.H., He K. (2020). Taxonomy and distribution of the genus *Typhlomys* in China based on karyotypic and phylogenetic analyses. Acta Theriol. Sin..

[9] Pu Y.T., Wan T., Fan R.H., Fu C.K., Tang K.Y., Jiang X.L., Zhang B.W., Hu T.L., Chen S.D., Liu S.Y. (2022). A new species of the genus *Typhlomys* Milne-Edwards, 1877 (Rodentia: Platacanthomyidae) from Chongqing, China. Zool. Res..

[10] Wu J.Y. (1990). The Discovery of Chinese Pygmy Dormouse (*Typhlomys cinereus*) in Qinling mountain. Zool. Res..

[11] Milne-Edwards A. (1877). Sur quelques mammifères et crustacés nouveaux. Bull. Soc Philomath. Paris.

[12] Hu T.L., Cheng F., Xu Z., Chen Z.Z., Zhang B.W. (2021). Molecular and morphological evidence for a new species of the genus *Typhlomys* (rodentia: Platacanthomyidae). Zool. Res..

[13] Ballard J.W., Whitlock M.C. (2004). The incomplete natural history of mitochondria. Mol. Ecol..

[14] Zhou Z.X., Liu B., Gong J., Bai Y.L., Yang J.Y., Xu P. (2020). Phylogeny and population genetics of species in *Takifugu* genus based on mitochondrial genome. J. Fish. China.

[15] Zhang W.P., Qiu B.Y., Zhang D.S. (2023). Adaptive Evolution Analysis of Mitochondrial Genomes in Anseriform Birds with Various Feeding Habits. Curr. Biotechnol..

[16] Hu T.W., Wang S.H., Zhang D.S. (2021). Adaptive Evolution of Mitochondrial Genome in Red-blooded Antarctic Fish and White-blooded Antarctic Fish. Genom. Appl. Biolo..

[17] Lv X., Cong H., Kong L., Motokawa M., Harada M., Wu Y., Li Y.C. (2016). The nearly complete mitochondrial genome of chinese pygmy dormouse *Typhlomys cinereus* (rodentia: Platacanthomyidae). Mitochondrial DNA Resour..

[18] Chen X., Ni G., He K., Ding Z.L., Zhang Y.P. (2018). Capture hybridization of long-range dna fragments for high-throughput sequencing. Methods. Mol. Biol..

[19] Yang M., Song L., Zhou L., Shi Y., Song N., Zhang Y. (2020). Mitochondrial genomes of four satyrine butterflies and phylogenetic relationships of the family *Nymphalidae* (Lepidoptera: Papilionoidea). Int. J. Biol. Macromol..

[20] Nicolas D., Patrick M., Guillaume S. (2017). Novoplasty: De novo assembly of organelle genomes from whole genome data. Nucleic. Acids. Res..

[21] Bernt M., Donath A., Juehling F., Stadler P. (2012). Mitos: Improved de novo metazoan mitochondrial genome annotation. Mol. Phylogenet. Evol..

[22] Tamura K., Stecher G., Kumar S. (2021). MEGA11: Molecular Evolutionary Genetics Analysis Version 11. Mol. Biol. Evol..

[23] Hassanin A., Leger N., Deutsch J. (2005). Evidence for multiple reversals of asymmetric mutational constraints during the evolution of the mitochondrial genome of Metazoa, and consequences for phylogenetic inferences. Syst. Biol..

[24] Rozas J., Ferrer-Mata A., Sánchez-DelBarrio J.C., Guirao-Rico S., Librado P., Ramos-Onsins S.E., Sánchez-Gracia A. (2017). DnaSP 6: DNA Sequence Polymorphism Analysis of Large Data Sets. Mol. Biol. Evol..

[25] Nguyen L.T., Schmidt H.A., von Haeseler A., Minh B.Q. (2015). IQ-TREE: A fast and effective stochastic algorithm for estimating maximum-likelihood phylogenies. Mol. Biol. Evol..

[26] Ronquist F., Teslenko M., Van Der Mark P., Ayres D.L., Darling A., Höhna S., Larget B., Liu L., Suchard M.A., Huelsenbeck J.P. (2012). MrBayes 3.2: Efficient Bayesian phylogenetic inference and model choice across a large model space. Syst Biol..

[27] Zhang D., Gao F.L., JakovliĆ I., Zou H., Zhang J., Li W.X., Wang G.T. (2020). PhyloSuite: An integrated and scalable desktop platform for streamlined molecular sequence data management and evolutionary phylogenetics studies. Mol. Ecol. Resour..

[28] Lanfear R., Frandse P.B., Wright A.M., Senfeld T., Calcott B. (2016). PartitionFinder 2: New Methods for Selecting Partitioned Models of Evolution for Molecular and Morphological Phylogenetic Analyses. Mol. Biol. Evol..

[29] Rambaut A. (2018). Figtree ver 1.4.4. Institute of Evolutionary Biology.

[30] Bouckaert R., Heled J., Kühnert D., Vaughan T., Wu C.H., Xie D., Suchard M.A.A., Rambaut A., Drummond A.J. (2014). BEAST 2: A software platform for Bayesian evolutionary analysis. PLoS Comput. Biol..

[31] Beard K.C., Qi T., Dawson M.R., Wang B., Li C. (1994). A diverse new primate fauna from middle eocene fissure-fillings in southeastern china. Nature.

[32] Andrew R., Drummond A.J., Dong X., Guy B., Marc A.S. (2018). Posterior Summarization in Bayesian Phylogenetics Using Tracer 1.7. Syst. Biol..

[33] Yu Y., Harris A.J., Blair C., He X. (2015). RASP (Reconstruct Ancestral State in Phylogenies): A tool for historical biogeography. Mol. Biol. Evol..

[34] Ree R.H., Smith S.A. (2008). Maximum likelihood inference of geographic range evolution by dispersal, local extinction, and cladogenesis. Syst. Biol..

[35] Ronquist F. (1997). Dispersal-vicariance analysis: A new approach to the quantification of historical biogeography. Syst. Biol..

[36] Landis M.J., Matzke N.J., Moore B.R., Huelsenbeck J.P. (2013). Bayesian analysis of biogeography when the number of areas is large. Syst. Biol..

[37] Wang X., Zhang H., Kitching I., Xu Z.B., Huang Y.X. (2021). First mitogenome of subfamily *langiinae* (lepidoptera: Sphingidae) with its phylogenetic implications. Gene.

[38] Minh B.Q., Nguyen M.A.T., Haeseler A.V. (2013). Ultrafast approximation for phylogenetic bootstrap. Mol. Biol. Evol..

[39] Da Fonseca R.R., Johnson W.E., O’Brien S.J., Ramos M.J., Antunes A. (2008). The adaptive evolution of the mammalian mitochondrial genome. BMC Genom..

[40] Zhang D.X., Szymura J.M., Hewitt G.M. (1995). Evolution and structural conservation of the control region of insect mitochondrial DNA. Mol. Evol..

[41] Árnadóttir E.R., Moore K.H.S., Guðmundsdóttir V.B., Ebenesersdóttir S.S., Guity K., Jónsson H., Helgason A. (2024). The rate and nature of mitochondrial DNA mutations in human pedigrees. Cell.

[42] Galtier N., Roux C., Rousselle M., Romiguier J., Figuet E., Glémin S., Bierne N., Duret L. (2018). Codon Usage Bias in Animals: Disentangling the Effects of Natural Selection, Effective Population Size, and GC-Biased Gene Conversion. Mol. Biol. Evol..

[43] Huang Y.X., Xing Z.P., Zhang H., Xu Z.B., Tao L.L., Hu H.Y., Kitching I.J., Wang X. (2022). Characterization of the Complete Mitochondrial Genome of Eight Diurnal Hawkmoths (Lepidoptera: Sphingidae): New Insights into the Origin and Evolution of Diurnalism in Sphingids. Insects.

[44] Zhang L., Sun K., Csorba G., Hughes A.C., Jin L., Xiao Y., Feng J. (2021). Complete mitochondrial genomes reveal robust phylogenetic signals and evidence of positive selection in horseshoe bats. BMC. Ecol. Evol..

[45] Wirth C., Brandt U., Hunte C., Zickermann V. (2016). Structure and function of mitochondrial complex I. Biochim. Biophy. Acta (BBA)-Bioenerg..

[46] Wang Y.L., Pan Z.Q., Chen K.K., Tao R.S., Su C.Y., Hao J.S., Yang Q. (2019). Genetic differentiation and phylogeography of the alpine butterfly Parnassius glacialis (Papilionidae: Parnassinae) in China: Evidence from mitogenomic AT-rich region. Acta Entomol. Sin..

