# Supplementary material for: Mitogenomic Insights into Phylogeny, Biogeography and Adaptive Evolution of the Genus Typhlomys (Rodentia: Platacanthomyidae)
Source: Animals (Basel). 2025 Sep 27;15(19):2823. doi: 10.3390/ani15192823 (PMC12523778; doi:10.3390/ani15192823)
Supplement: Supplementary file 1 [file animals-15-02823-s001.zip › animals-3861371-supplementary-file s1.pdf]

**Supplementary File S1**

# Mitogenomic Insights into Phylogeny, Biogeography and Adaptive Evolution of the Genus *Typhlomys* (Rodentia: Platacanthomyidae)

**Chao Na [1], Xiaohan Wang [1], Yaxin Cheng [1], Yixin Huang [1], Shuiwang He [2], Laxman Khanal [3,4], Shunde Chen [5], Xuelong Jiang [2,*] and Zhongzheng Chen [1,*]**

1 Collaborative Innovation Center of Recovery and Reconstruction of Degraded Ecosystem in Wanjiang Basin Co-Founded by Anhui Province and Ministry of Education, School of Ecology and Environment, Anhui Normal University, Wuhu 241002, China; n1525620041@163.com (C.N.); wx374989939@163.com (X.W.); yaxin0701@ahnu.edu.cn (Y.C.); huangyx@ahnu.edu.cn (Y.H.)

2 State Key Laboratory of Genetic Resources and Evolution, Yunnan Key Laboratory of Biodiversity and Ecological Conservation of Gaoligong Mountain, Kunming Institute of Zoology, Chinese Academy of Sciences, Kunming 650204, China; heshuiwang@mail.kiz.ac.cn

3 Central Department of Zoology, Institute of Science and Technology, Tribhuvan University, Kathmandu 44618, Nepal; khanal@ccdz.tu.edu.np

4 International Centre for Biodiversity and Primates Conservation, Dali University, Dali 671003, China

5 College of Life Sciences, Sichuan Normal University, Chengdu 610101, China; csd111@126.com

* Correspondence: jiangxl@mail.kiz.ac.cn (X.J.); chenzz@ahnu.edu.cn (Z.C.)

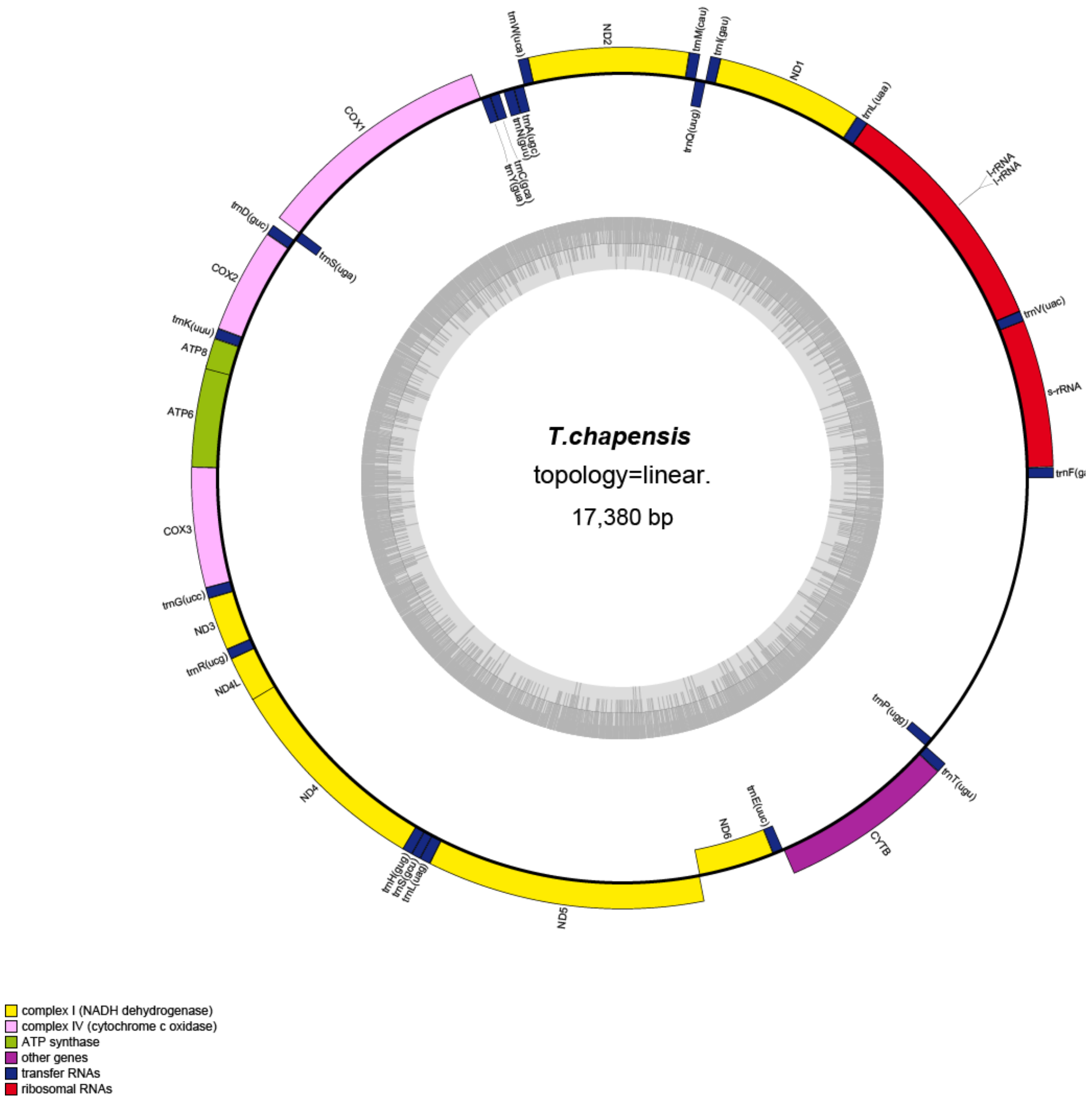


Figure S1. Circular maps of the mitochondrial genome *T.chapensis* (098564).

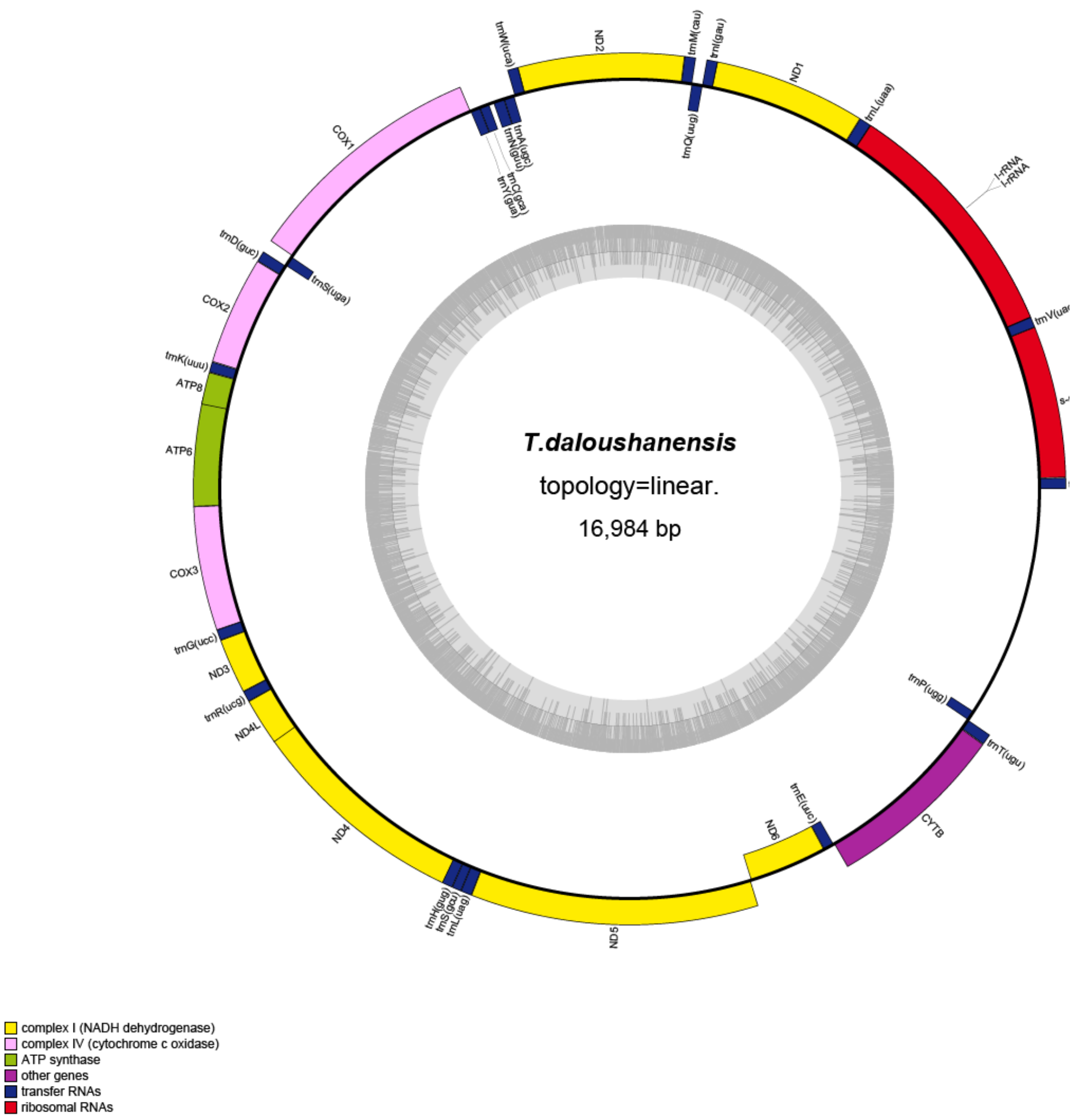


Figure S2. Circular maps of the mitochondrial genome *T.daloushanensis*(Y204005).

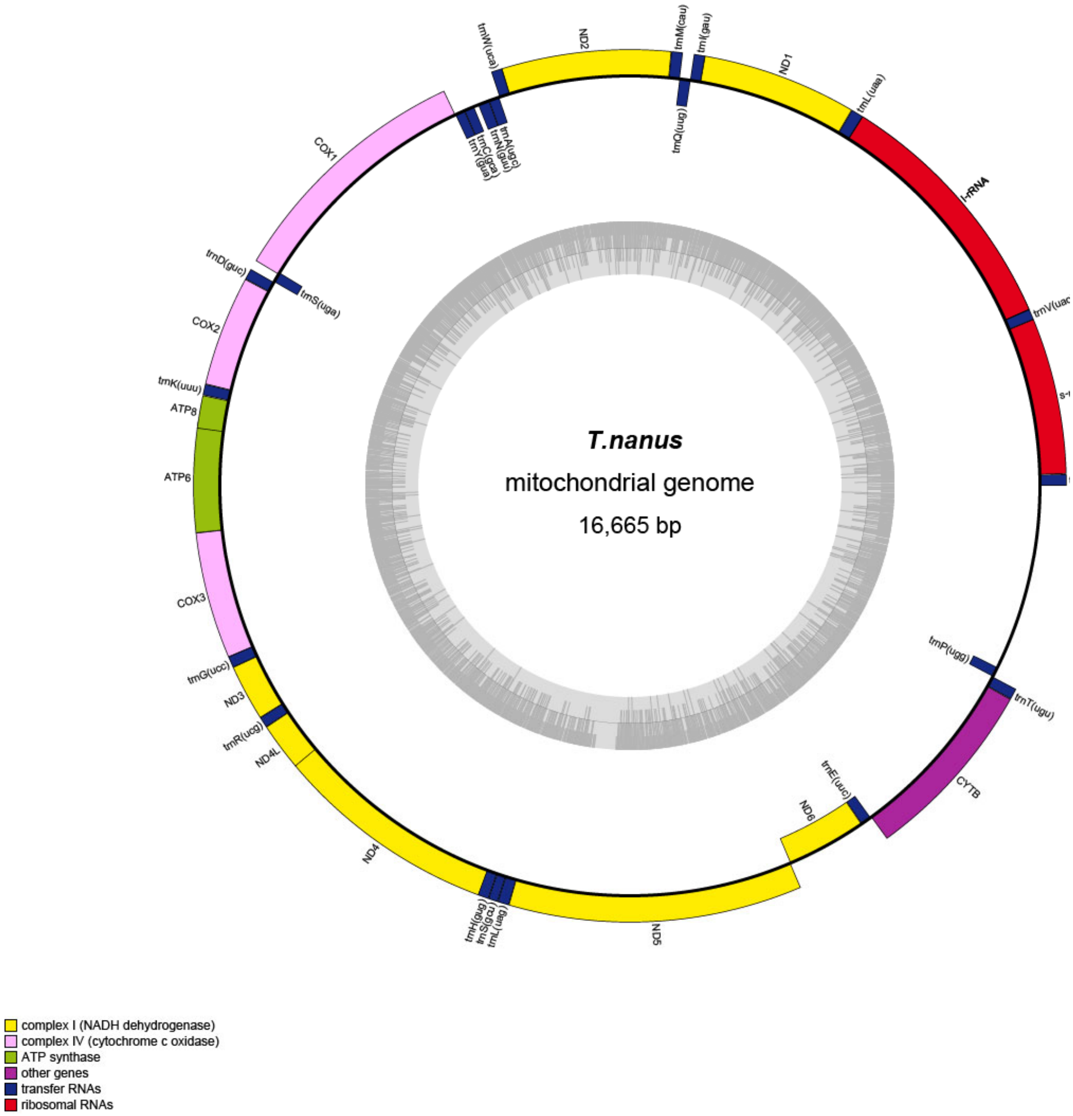


Figure S3. Circular maps of the mitochondrial genome *T.nanus*.

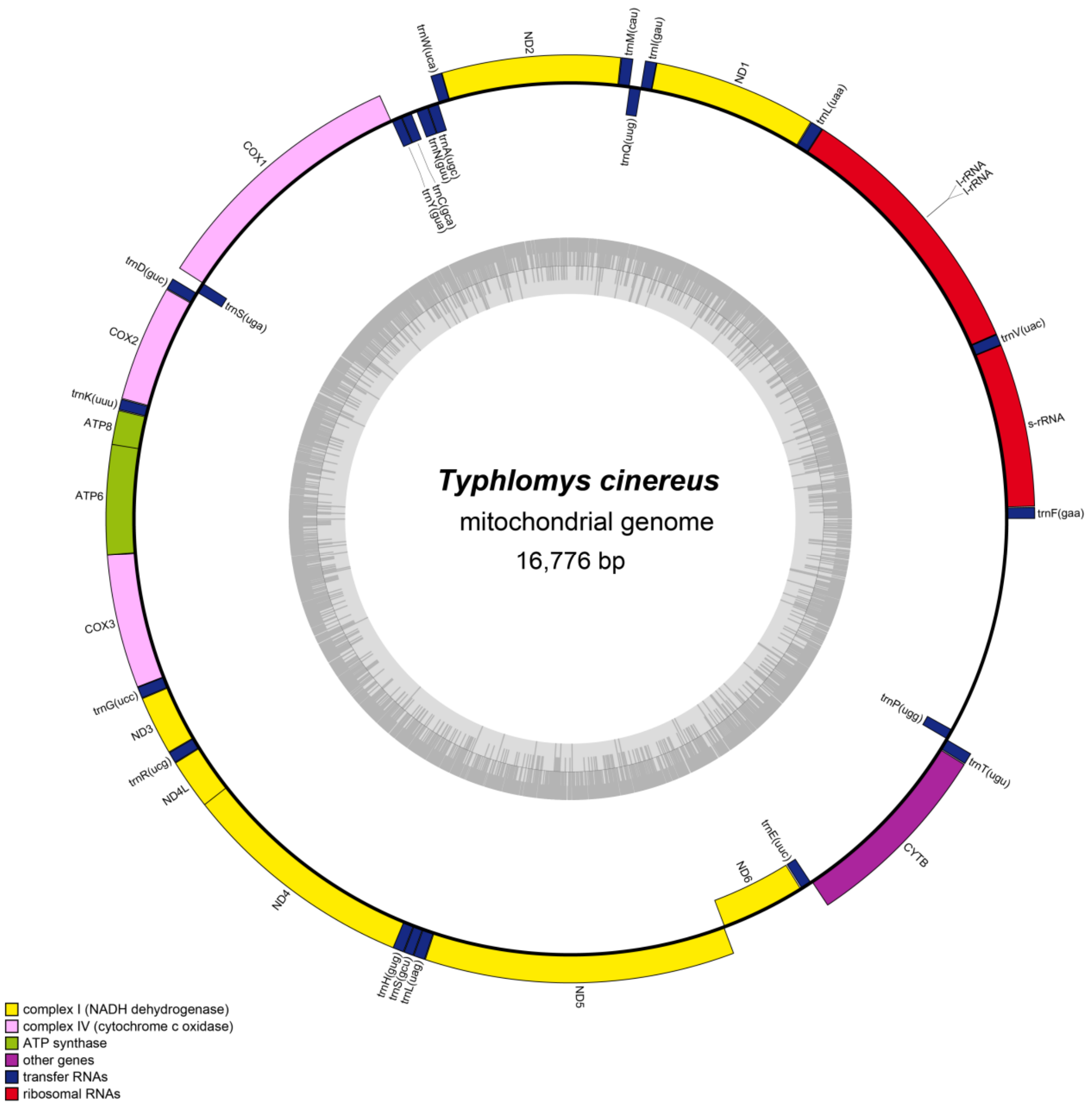


Figure S4. Circular maps of the mitochondrial genome *T.cinereus*(Z201312257).

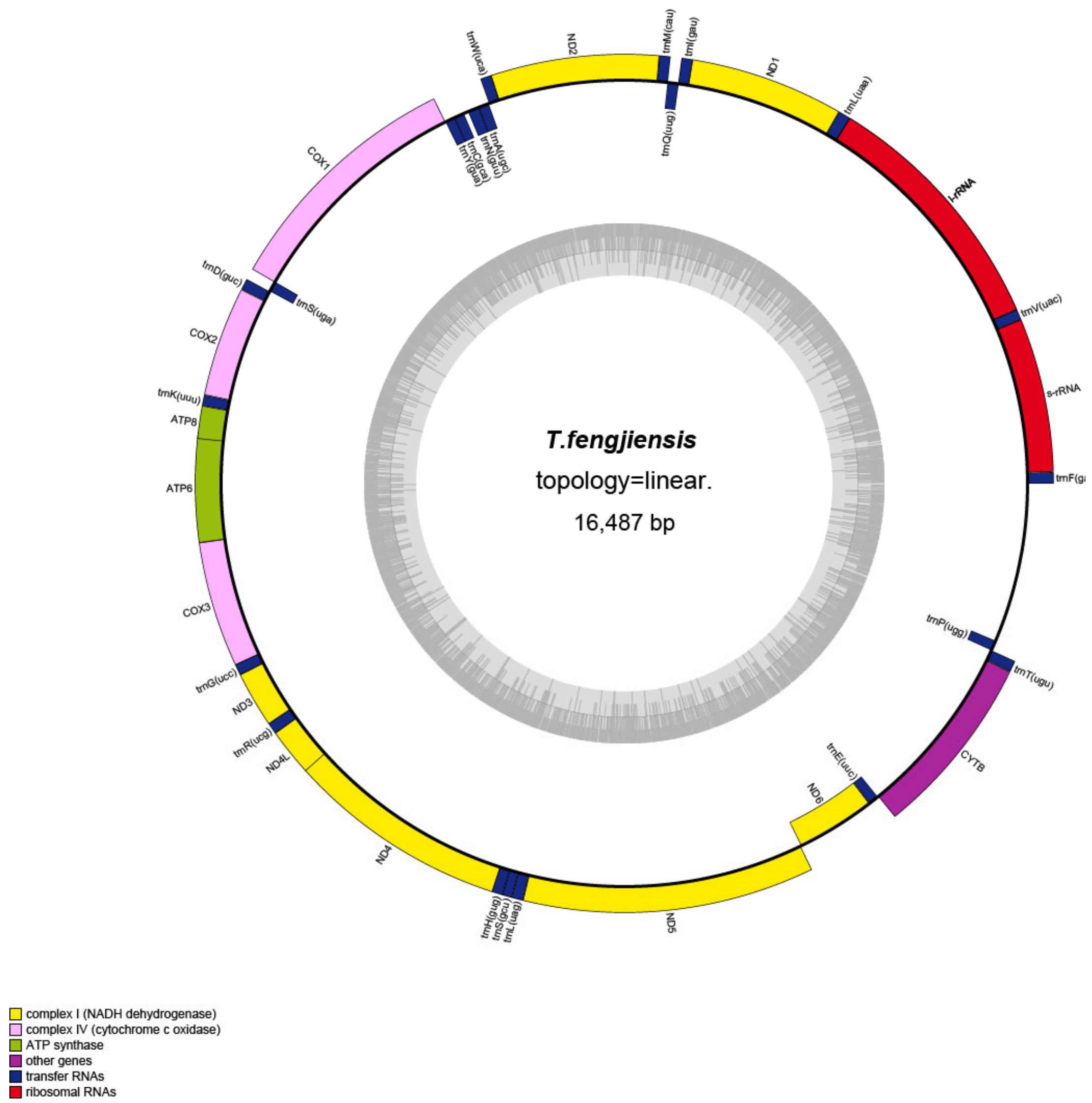


Figure S5. Circular maps of the mitochondrial genome *T.fengjiensis*.

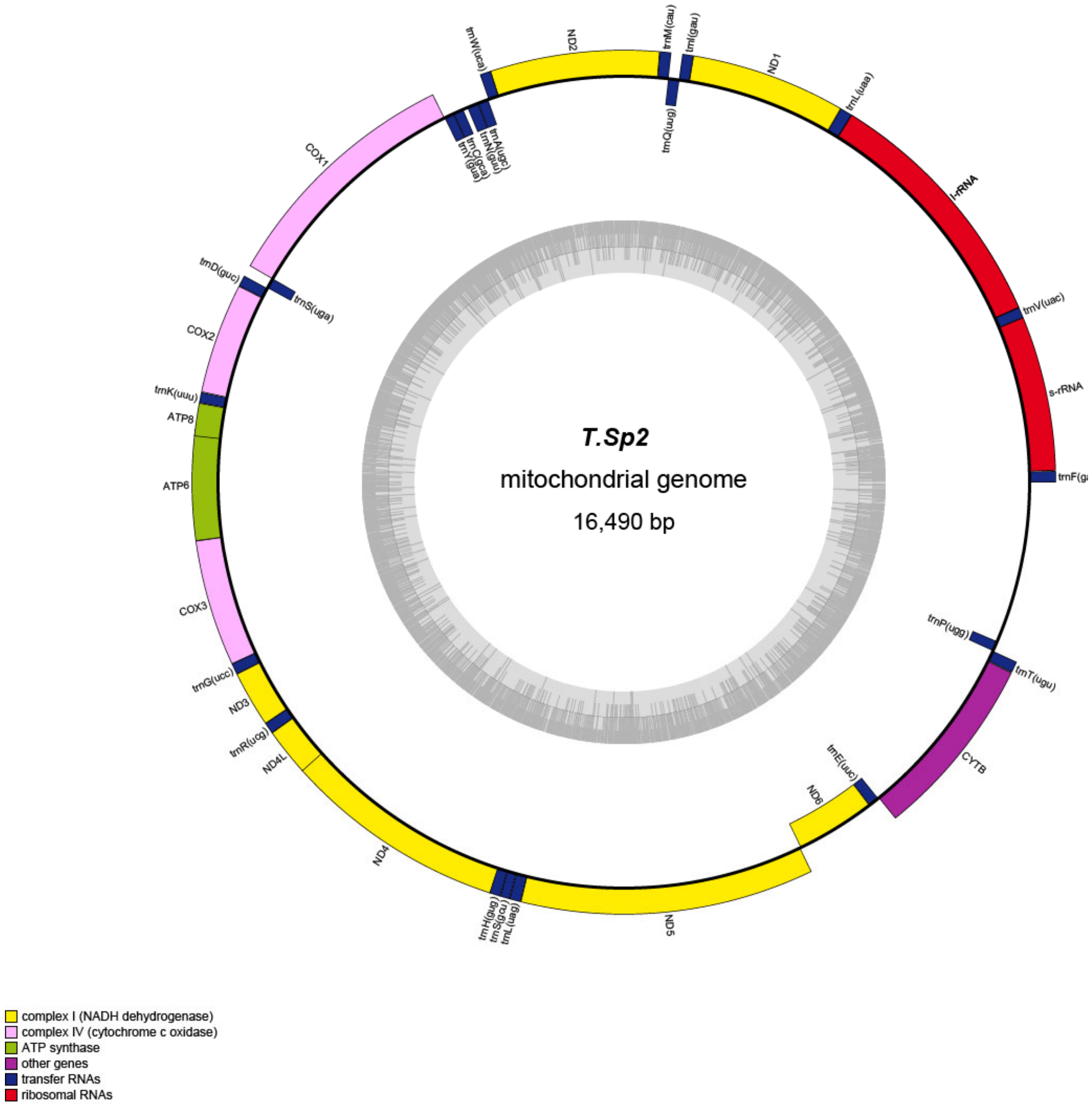


Figure S6. Circular maps of the mitochondrial genome *T.* sp. 2

## 3.3 Ribosomal RNA and transfer RNA

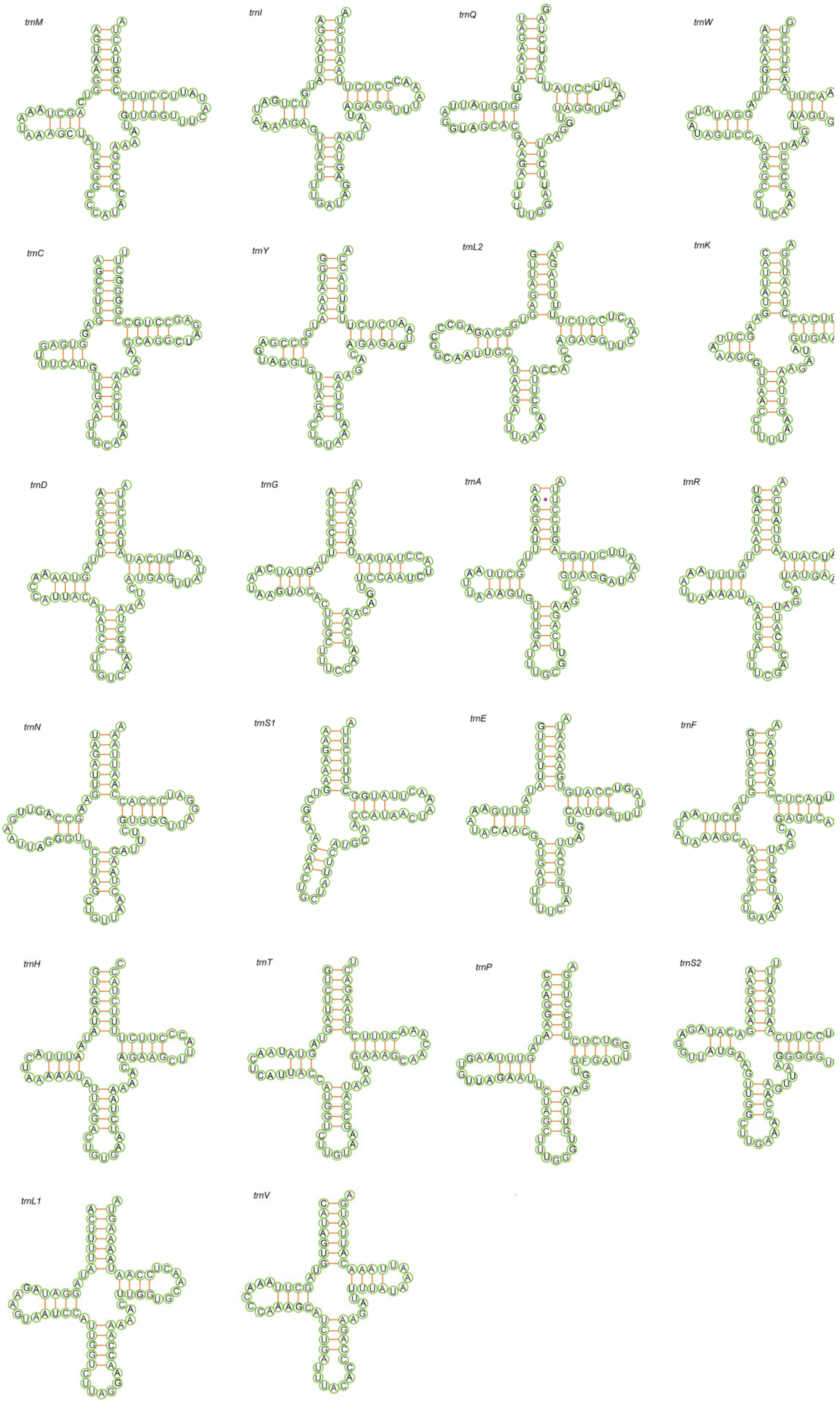


Figure S7. Predicted secondary cloverleaf structure for the tRNAs of *T. cinereus* (Z201312257).

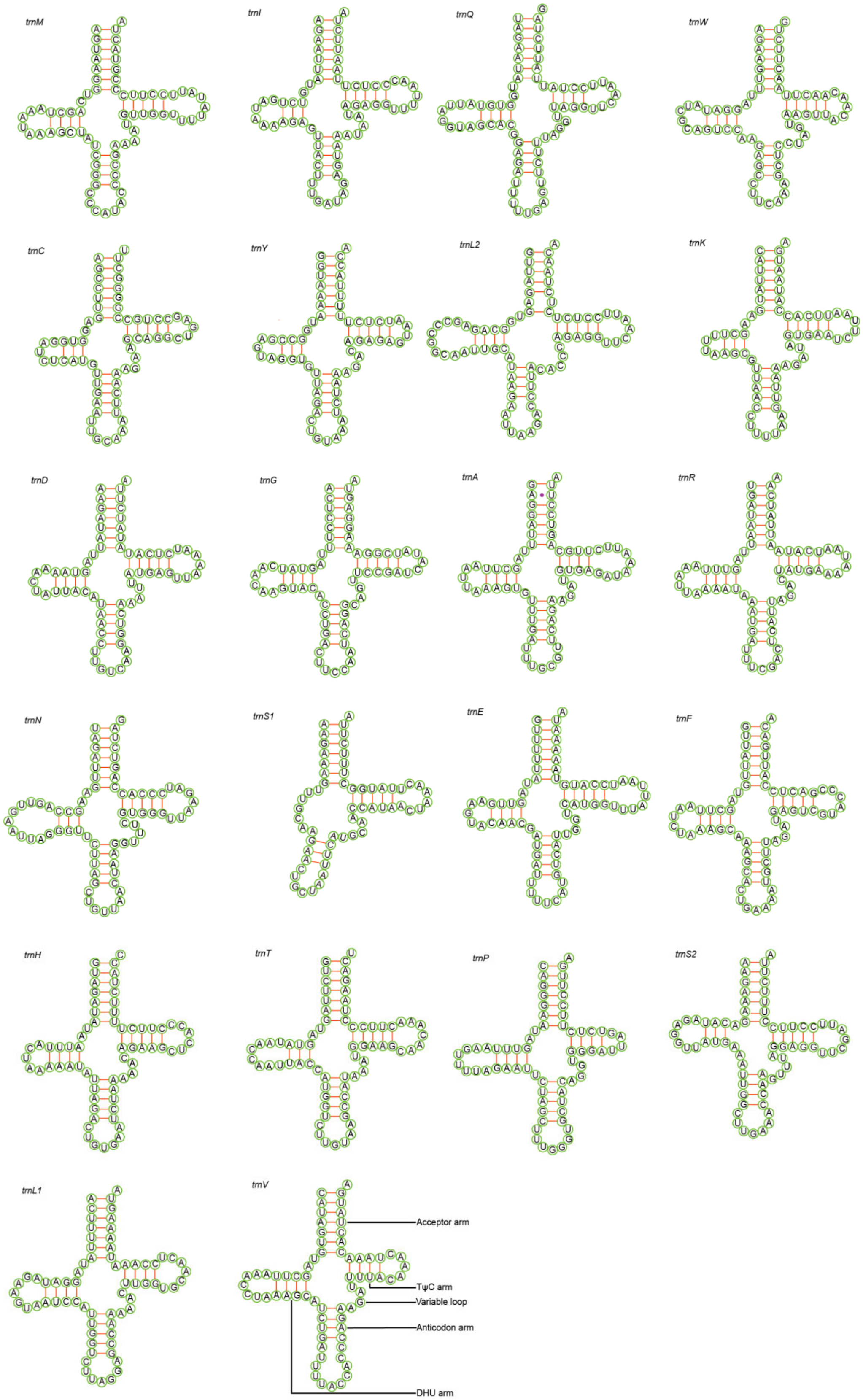


Figure S8. Predicted secondary cloverleaf structure for the tRNAs of *T. daloushanensis* (Y204005).

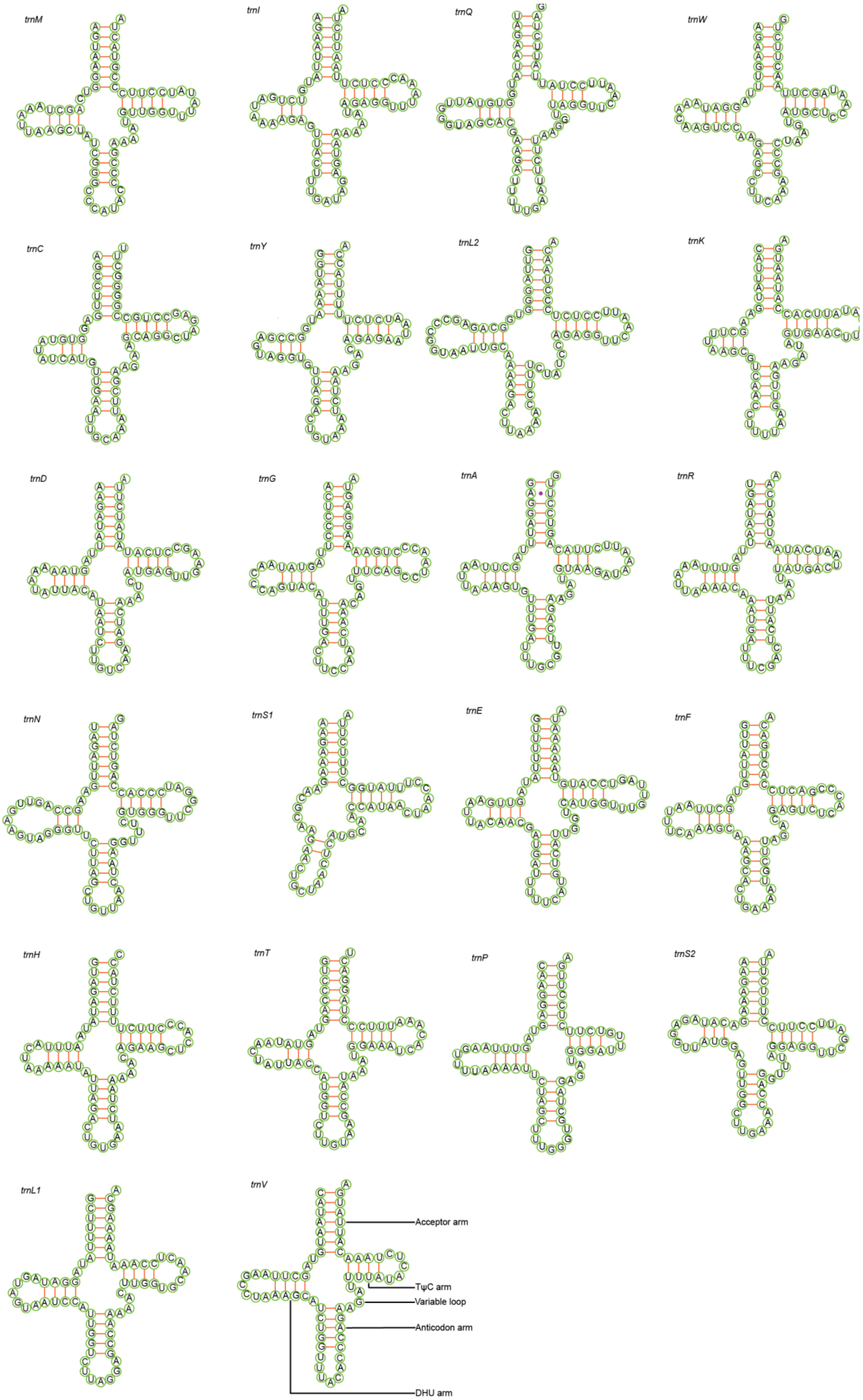


Figure S9. Predicted secondary cloverleaf structure for the tRNAs of *T. chapensis* (098564).

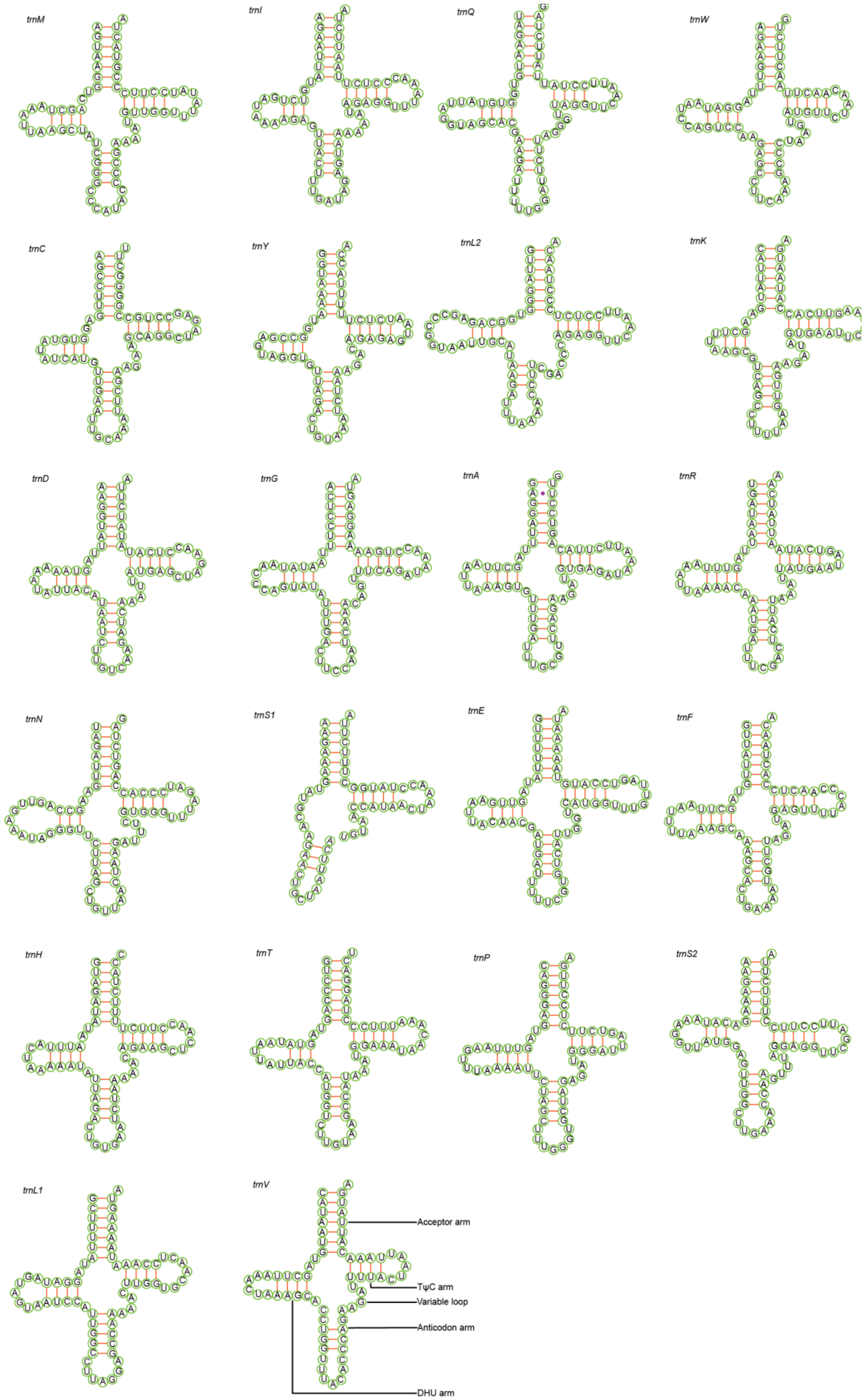


Figure S10. Predicted secondary cloverleaf structure for the tRNAs of *T. nanus* (0811188).

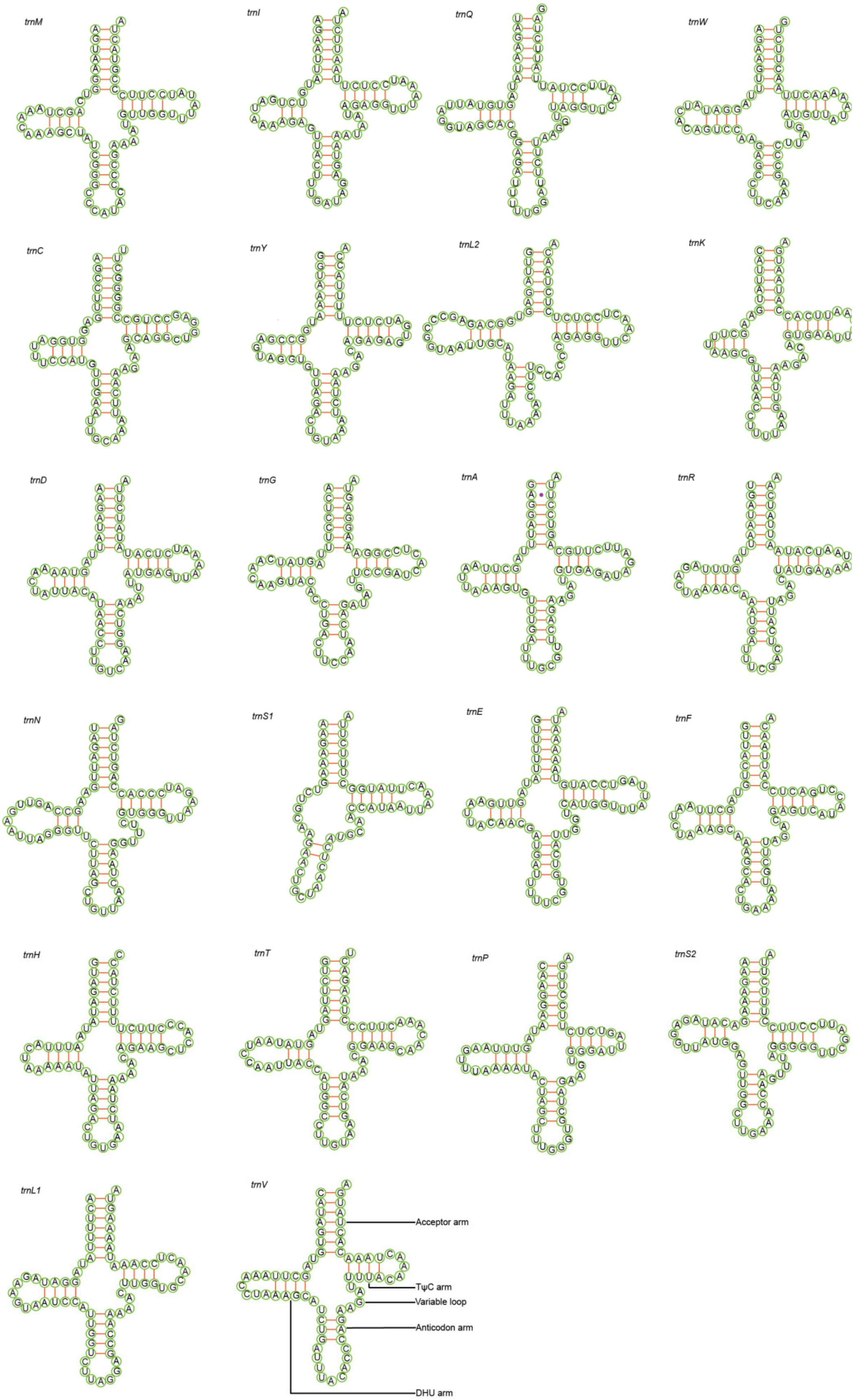


Figure S11. Predicted secondary cloverleaf structure for the tRNAs of *T.* sp. 2 (1503001).

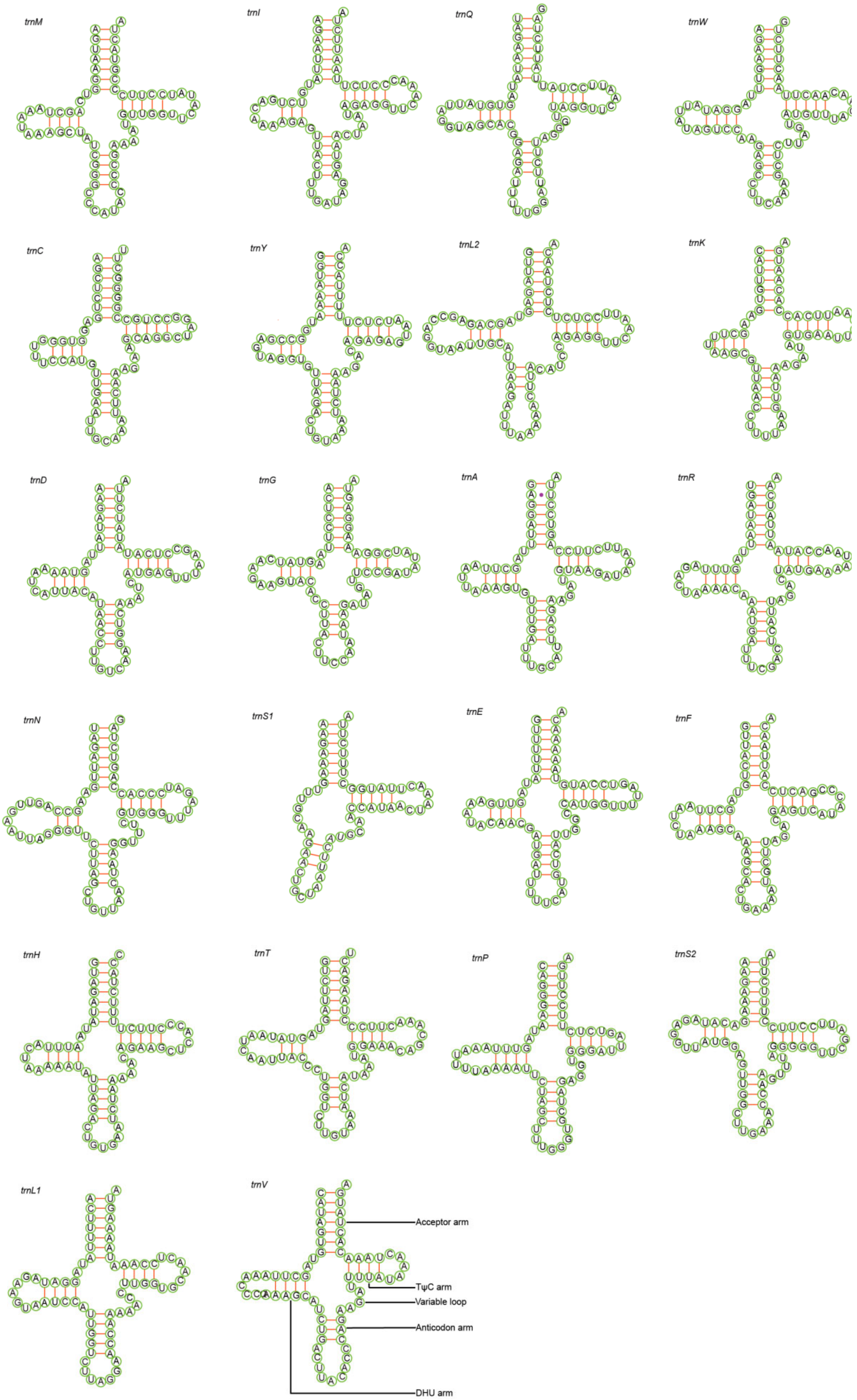


Figure S12. Predicted secondary cloverleaf structure for the tRNAs of *T. fengjiensis* (csd4273).

## 3.3 Ribosomal RNA and transfer RNA.

Table S1 Nucleotide composition of separate regions on mitogenome of *T.daloushanensis* (Y204005).

| *T.daloushanensis* (Y204005). | | | | | | | | | |
|---|---|---|---|---|---|---|---|---|---|
| Regions | Size (bp) | A(%) | C(%) | G(%) | T(%) | A+T(%) | C+G(%) | AT-skew | GC-skew |
| Mitochondrial genome | 16984 | 34.2 | 25.3 | 11.8 | 28.7 | 62.9 | 37.1 | 8.74 | -36.4 |
| PCGs | 11390 | 32.5 | 27.3 | 10.7 | 29.4 | 62.0 | 38.0 | 5.00 | -43.7 |
| PCGs(J) | 10868 | 32.1 | 27.2 | 10.9 | 29.8 | 62.0 | 38.0 | 3.71 | -42.9 |
| PCGs(N) | 522 | 40.6 | 30.7 | 6.7 | 22.0 | 62.6 | 37.4 | 29.7 | -64.2 |
| tRNAs | 1507 | 35.7 | 20.7 | 14.5 | 29.1 | 64.8 | 35.2 | 10.2 | -17.6 |
| tRNAs(J) | 957 | 36.4 | 18.8 | 15.3 | 29.6 | 65.9 | 34.1 | 10.3 | -10.3 |
| tRNAs(N) | 550 | 34.5 | 24.0 | 13.3 | 28.2 | 62.7 | 37.3 | 10.1 | -28.7 |
| rRNAs | 2528 | 38.4 | 20.4 | 15.9 | 25.4 | 63.7 | 36.3 | 20.4 | -15.4 |
| A+T | 1005 | 37.6 | 24.2 | 11.1 | 27.1 | 64.7 | 35.3 | / | / |

Table S2 Nucleotide composition of separate regions on mitogenome of *T.chapensis* (098564)

| *T.chapensis* (098564) | | | | | | | | | |
|---|---|---|---|---|---|---|---|---|---|
| Regions | Size(bp) | A(%) | C(%) | G(%) | T(%) | A+T(%) | C+G(%) | AT-skew | GC-skew |
| Mitochondrial genome | 17380 | 33.5 | 26.8 | 12.6 | 27.2 | 60.6 | 39.4 | 10.4 | -36.0 |
| PCGs | 11422 | 32.1 | 29.1 | 11.3 | 27.6 | 59.7 | 40.3 | 7.54 | -44.2 |
| PCGs(J) | 10897 | 31.8 | 28.9 | 11.5 | 27.9 | 59.6 | 40.4 | 6.54 | -43.1 |
| PCGs(N) | 525 | 39.2 | 32.8 | 7.0 | 21.0 | 60.2 | 39.8 | 30.2 | -64.8 |
| tRNAs | 1510 | 35.6 | 21.2 | 14.2 | 29.0 | 64.6 | 35.4 | 10.2 | -19.8 |
| tRNAs(J) | 959 | 36.0 | 19.5 | 14.9 | 29.6 | 65.6 | 34.4 | 9.76 | -13.4 |
| tRNAs(N) | 551 | 35.0 | 24.1 | 12.9 | 27.9 | 63.0 | 37.0 | 11.27 | -30.3 |
| rRNAs | 2537 | 37.9 | 21.7 | 16.6 | 23.8 | 61.6 | 38.4 | 22.89 | -13.3 |

| A+T | 820 | 33.9 | 26.3 | 11.5 | 28.3 | 62.2 | 37.8 | / | / |
|---|---|---|---|---|---|---|---|---|---|

Table S3 Nucleotide composition of separate regions on mitogenome of *T.nanus* (0811188)

| *T.nanus* (0811188) | | | | | | | | | |
|---|---|---|---|---|---|---|---|---|---|
| Regions | Size(bp) | A(%) | C(%) | G(%) | T(%) | A+T(%) | C+G(%) | AT-skew | GC-skew |
| Mitochondrial genome | 16665 | 33.9 | 25.9 | 12.3 | 28.0 | 61.8 | 38.2 | 9.55 | -35.6 |
| PCGs | 11423 | 32.5 | 27.8 | 11.1 | 28.6 | 61.1 | 38.9 | 6.38 | -42.9 |
| PCGs(J) | 10898 | 32.1 | 27.6 | 11.3 | 29.0 | 61.1 | 38.9 | 5.07 | -41.9 |
| PCGs(N) | 525 | 39.8 | 32.4 | 6.9 | 21.0 | 60.8 | 39.2 | 30.9 | -65.1 |
| tRNAs | 1507 | 35.8 | 21.2 | 13.9 | 29.1 | 64.9 | 35.1 | 10.3 | -20.8 |
| tRNAs(J) | 956 | 36.5 | 18.6 | 14.7 | 30.1 | 66.6 | 33.4 | 9.61 | -11.7 |
| tRNAs(N) | 551 | 34.5 | 25.6 | 12.5 | 27.4 | 61.9 | 38.1 | 11.5 | -34.4 |
| rRNAs | 2540 | 38.2 | 21.4 | 16.4 | 24.0 | 62.2 | 37.8 | 22.8 | -13.2 |
| A+T | 1005 | 37.6 | 24.2 | 11.1 | 27.1 | 64.7 | 35.3 | / | / |

Table S4 Nucleotide composition of separate regions on mitogenome of *T.fengjiensis* (csd4273)

| *T.fengjiensis*(csd4273) | | | | | | | | | |
|---|---|---|---|---|---|---|---|---|---|
| Regions | Size(bp) | A(%) | C(%) | G(%) | T(%) | A+T(%) | C+G(%) | AT-skew | GC-skew |
| Mitochondrial genome | 16487 | 34.5 | 24.5 | 11.7 | 29.3 | 63.8 | 36.2 | 8.2 | -35.4 |
| PCGs | 11402 | 33.0 | 26.2 | 10.5 | 30.2 | 63.3 | 36.7 | 4.4 | -42.8 |
| PCGs(J) | 10880 | 32.7 | 26.0 | 10.7 | 30.6 | 63.3 | 36.7 | 3.3 | -41.7 |
| PCGs(N) | 522 | 41.0 | 30.8 | 6.3 | 21.8 | 62.8 | 37.2 | 30.6 | -65.9 |
| tRNAs | 1506 | 36.1 | 20.7 | 14.2 | 29.1 | 65.1 | 34.9 | 10.8 | -18.6 |
| tRNAs(J) | 956 | 37.0 | 18.5 | 14.6 | 29.8 | 66.8 | 33.2 | 10.8 | -11.8 |
| tRNAs(N) | 550 | 34.4 | 24.4 | 13.5 | 27.8 | 62.2 | 37.8 | 10.6 | -28.8 |
| rRNAs | 2533 | 38.2 | 19.9 | 16.1 | 25.9 | 64.1 | 35.9 | 19.2 | -10.6 |

| | | | | | | | | | |
|---|---|---|---|---|---|---|---|---|---|
| A+T | 625 | 34.4 | 22.1 | 12.8 | 30.7 | 65.1 | 34.9 | / | / |

Table S5 Nucleotide composition of separate regions on mitogenome of *T. cinereus* (Z201312257)

| Regions | Size(bp) | A(%) | C(%) | G(%) | T(%) | A+T(%) | C+G(%) | AT-skew | GC-skew |
|---|---|---|---|---|---|---|---|---|---|
| Mitochondrial genome | 16776 | 34.7 | 24.1 | 11.3 | 29.9 | 64.6 | 35.4 | 7.4 | -36.2 |
| PCGs | 11405 | 33.0 | 25.9 | 10.4 | 30.7 | 63.8 | 36.2 | 3.6 | -42.8 |
| PCGs(J) | 10880 | 32.7 | 25.7 | 10.6 | 31.1 | 63.8 | 36.2 | 2.5 | -41.7 |
| PCGs(N) | 525 | 40.6 | 29.9 | 6.3 | 23.2 | 63.8 | 36.2 | 27.3 | -65.2 |
| tRNAs | 1507 | 36.5 | 20.4 | 13.8 | 29.3 | 65.8 | 34.2 | 10.9 | -19.6 |
| tRNAs(J) | 957 | 37.4 | 18.3 | 14.3 | 30.0 | 67.4 | 32.6 | 11.0 | -12.3 |
| tRNAs(N) | 550 | 34.9 | 24.0 | 12.9 | 28.2 | 63.1 | 36.9 | 10.6 | -30.1 |
| rRNAs | 2525 | 38.6 | 19.3 | 15.8 | 26.3 | 64.9 | 35.1 | 19.0 | -10.0 |
| A+T | 1062 | 37.6 | 23.0 | 9.4 | 30.0 | 67.6 | 32.4 | / | / |

Table S6 Nucleotide composition of separate regions on mitogenome of *T.* sp. 2 (1503001)

T. sp. 2 (1503001)

| Regions | Size(bp) | A(%) | C(%) | G(%) | T(%) | A+T(%) | C+G(%) | AT-skew | GC-skew |
|---|---|---|---|---|---|---|---|---|---|
| Mitochondrial genome | 16490 | 34.3 | 24.7 | 11.8 | 29.9 | 63.5 | 36.5 | 6.9 | -35.3 |
| PCGs | 11402 | 32.8 | 36.4 | 10.7 | 30.2 | 62.9 | 37.1 | 4.1 | -42.3 |
| PCGs(J) | 10880 | 32.4 | 26.2 | 10.9 | 30.5 | 62.9 | 37.1 | 3.0 | -41.2 |
| PCGs(N) | 522 | 40.8 | 29.7 | 6.9 | 22.6 | 63.4 | 36.6 | 28.7 | -62.3 |
| tRNAs | 1514 | 36.2 | 20.9 | 14.2 | 28.8 | 64.9 | 35.1 | 11.4 | -19.1 |
| tRNAs(J) | 957 | 36.9 | 19.0 | 14.7 | 29.4 | 66.2 | 33.8 | 11.3 | -12.7 |
| tRNAs(N) | 553 | 34.4 | 25.0 | 13.2 | 27.5 | 61.8 | 38.2 | 11.2 | -30.9 |
| rRNAs | 2528 | 38.6 | 19.7 | 15.8 | 25.9 | 64.5 | 35.5 | 19.7 | -11.0 |
| A+T | 1059 | 37.5 | 22.8 | 10.8 | 29.0 | 66.5 | 33.5 | / | / |

## 3.4 Overlapping and intergenic spacer regions

Table S7 Mitogenomic organization of *T. daloushanensis* (Y204005)

| | Position | | Strand | Length | Intergenic | Codons | |
|---|---|---|---|---|---|---|---|
| | From | To | | | nucleotides | Start | Stop |
| trnF | 1 | 69 | + | 69 | 2 | | |
| rrnS | 72 | 1028 | + | 957 | -1 | | |
| trnV | 1028 | 1094 | + | 67 | 0 | | |
| rrnL | 1095 | 2665 | + | 1571 | 0 | | |
| trnL2 | 2666 | 2740 | + | 75 | 1 | | |
| ND1 | 2742 | 3698 | + | 957 | -2 | GTG | TAG |
| trnI | 3697 | 3765 | + | 69 | -3 | | |
| trnQ | 3763 | 3834 | - | 72 | -1 | | |
| trnM | 3834 | 3903 | + | 70 | 0 | | |
| ND2 | 3904 | 4947 | + | 1044 | -2 | ATA | TAG |
| trnW | 4946 | 5013 | + | 68 | 5 | | |
| trnA | 5019 | 5087 | - | 69 | 0 | | |
| trnN | 5088 | 5161 | - | 74 | 2 | | |
| trnC | 5194 | 5258 | - | 65 | 0 | | |
| trn | 5259 | 5322 | - | 64 | 1 | | |
| COX1 | 5324 | 6856 | + | 1533 | 8 | ATG | TAA |
| trnS2 | 6865 | 6933 | - | 69 | 3 | | |
| trnD | 6937 | 7005 | + | 69 | 1 | | |
| COX2 | 7007 | 7690 | + | 684 | 2 | ATG | TAA |
| trnK | 7693 | 7758 | + | 66 | 1 | | |
| atp8 | 7760 | 7963 | + | 204 | -43 | ATG | TAA |
| atp6 | 7921 | 8601 | + | 681 | -1 | ATG | TAA |
| COX3 | 8601 | 9385 | + | 785 | -1 | ATG | TTA |
| trnG | 9385 | 9452 | + | 68 | 0 | | |
| ND3 | 9453 | 9800 | + | 348 | 0 | ATC | TAA |
| trnR | 9801 | 9869 | + | 69 | 0 | | |
| ND4L | 9870 | 10166 | + | 297 | -7 | ATG | TAA |
| ND4 | 10160 | 11537 | + | 1383 | 0 | ATG | ACT |
| trnH | 11538 | 11605 | + | 68 | 0 | | |
| trnS1 | 11606 | 11664 | + | 59 | 0 | | |
| trnL1 | 11665 | 11734 | + | 70 | 0 | | |
| ND5 | 11735 | 13546 | + | 1812 | 5 | ATT | TAA |
| ND6 | 13552 | 14073 | - | 522 | 1 | CTA | CAT |
| trnE | 14075 | 14145 | - | 71 | 5 | | |
| Cytb | 14151 | 15290 | + | 1140 | 2 | ATG | AGA |
| trnT | 15293 | 15362 | + | 70 | 1 | | |
| trnP | 15364 | 15429 | - | 66 | 335 | | |

Table S8 Mitogenomic organization of *T. chapensis* (098564)

| | Position | | Strand | Length | Intergenic nucleotides | Codons | |
|---|---|---|---|---|---|---|---|
| | From | To | | | | Start | Stop |
| trnF | 1 | 70 | + | 70 | 2 | | |
| rrnS | 73 | 1034 | + | 962 | -1 | | |
| trnV | 1034 | 1100 | + | 67 | 0 | | |
| rrnL | 1101 | 2675 | + | 1575 | 0 | | |
| trnL2 | 2676 | 2750 | + | 75 | 1 | | |
| ND1 | 2752 | 3708 | + | 957 | -2 | GTG | TAG |
| trnI | 3707 | 3775 | + | 69 | -3 | | |
| trnQ | 3773 | 3844 | - | 72 | -1 | | |
| trnM | 3844 | 3913 | + | 70 | 0 | | |
| ND2 | 3914 | 4957 | + | 1044 | -2 | ATT | TAG |
| trnW | 4956 | 5023 | + | 68 | 4 | | |
| trnA | 5028 | 5096 | - | 69 | 0 | | |
| trnN | 5097 | 5170 | - | 74 | 2 | | |
| trnC | 5202 | 5267 | - | 66 | 0 | | |
| trnY | 5268 | 5331 | - | 64 | 1 | | |
| COX1 | 5333 | 6895 | + | 1563 | -13 | ATG | AGG |
| trnS2 | 6883 | 6951 | - | 69 | 3 | | |
| trnD | 6955 | 7023 | + | 69 | 1 | | |
| COX2 | 7025 | 7708 | + | 684 | 1 | ATG | TAG |
| trnK | 7710 | 7776 | + | 67 | 1 | | |
| atp8 | 7778 | 7981 | + | 204 | -43 | ATG | TAA |
| atp6 | 7939 | 8619 | + | 681 | -1 | ATG | TAA |
| COX3 | 8619 | 9403 | + | 785 | -1 | ATG | TTA |
| trnG | 9403 | 9472 | + | 70 | 0 | | |
| ND3 | 9473 | 9820 | + | 348 | 0 | ATA | TAA |
| trnR | 9821 | 9887 | + | 67 | 0 | | |
| ND4L | 9888 | 10184 | + | 297 | -7 | ATG | TAA |
| ND4 | 10178 | 11554 | + | 1270 | 0 | ATG | ACT |
| trnH | 11555 | 11622 | + | 68 | 0 | | |
| trnS1 | 11623 | 11682 | + | 60 | 0 | | |
| trnL1 | 11683 | 11752 | + | 70 | 0 | | |
| ND5 | 11753 | 13564 | + | 1812 | 1 | ATT | TAA |
| ND6 | 13566 | 14090 | - | 525 | 1 | CCT | CAT |
| trnE | 14092 | 14162 | - | 71 | 5 | | |
| Cytb | 14168 | 15307 | + | 1140 | 1 | ATG | AGA |
| trnT | 15309 | 15377 | + | 69 | 2 | | |
| trnP | 15380 | 15445 | - | 66 | 84 | | |

Table S9 Mitogenomic organization of *T. nanus* (0811188)

| | Position | | Strand | Length | Intergenic nucleotides | Codons | |
|---|---|---|---|---|---|---|---|
| | From | To | | | | Start | Stop |
| trnF | 1 | 70 | + | 70 | 2 | | |

| | | | | | | | |
|---|---|---|---|---|---|---|---|
| rrnS | 73 | 1038 | + | 966 | -1 | | |
| trnV | 1038 | 1103 | + | 66 | 0 | | |
| rrnL | 1104 | 2675 | + | 1572 | 0 | | |
| trnL2 | 2676 | 2750 | + | 75 | 1 | | |
| ND1 | 2752 | 3708 | + | 957 | -2 | GTG | TAG |
| trnI | 3707 | 3775 | + | 69 | -3 | | |
| trnQ | 3773 | 3844 | - | 72 | -1 | | |
| trnM | 3844 | 3913 | + | 70 | 0 | | |
| ND2 | 3914 | 4957 | + | 1044 | -2 | ATT | TAG |
| trnW | 4956 | 5023 | + | 68 | 4 | | |
| trnA | 5028 | 5096 | - | 69 | 0 | | |
| trnN | 5097 | 5170 | - | 74 | 2 | | |
| trnC | 5202 | 5267 | - | 66 | 0 | | |
| trnY | 5268 | 5331 | - | 64 | 1 | | |
| COX1 | 5333 | 6895 | + | 1563 | -13 | ATG | AGG |
| trnS2 | 6883 | 6951 | - | 69 | 3 | | |
| trnD | 6955 | 7023 | + | 69 | 1 | | |
| COX2 | 7025 | 7708 | + | 684 | 1 | ATG | TAA |
| trnK | 7710 | 7776 | + | 67 | 2 | | |
| atp8 | 7779 | 7982 | + | 204 | -43 | ATG | TAA |
| atp6 | 7940 | 8620 | + | 681 | -1 | ATG | TAA |
| COX3 | 8620 | 9404 | + | 785 | -1 | ATG | TTA |
| trnG | 9404 | 9472 | + | 69 | 0 | | |
| ND3 | 9473 | 9820 | + | 348 | 0 | ATA | TAA |
| trnR | 9821 | 9887 | + | 67 | 0 | | |
| ND4L | 9888 | 10184 | + | 297 | -7 | ATG | TAA |
| ND4 | 10178 | 11555 | + | 1383 | 0 | ATG | ACT |
| trnH | 11556 | 11623 | + | 68 | 0 | | |
| trnS1 | 11624 | 11682 | + | 59 | 0 | | |
| trnL1 | 11683 | 11752 | + | 70 | 0 | | |
| ND5 | 11753 | 13564 | + | 1812 | 1 | ATA | TAG |
| ND6 | 13566 | 14090 | - | 525 | 1 | CCT | CAT |
| trnE | 14092 | 14162 | - | 71 | 5 | | |
| Cytb | 14168 | 15307 | + | 1140 | 1 | ATG | AGA |
| trnT | 15309 | 15377 | + | 69 | 2 | | |
| trnP | 15380 | 15445 | - | 66 | 105 | | |

Table S10 Mitogenomic organization of *T. fengjiensis* (csd4273)

| | Position | | Strand | Length | Intergenic | Codons | |
|---|---|---|---|---|---|---|---|
| | From | To | | | nucleotides | Start | Stop |
| trnF | 1 | 69 | + | 69 | 2 | | |
| rrnS | 72 | 1029 | + | 958 | -1 | | |
| trnV | 1029 | 1095 | + | 67 | 0 | | |
| rrnL | 1096 | 2670 | + | 1575 | 0 | | |

| | | | | | | | |
|---|---|---|---|---|---|---|---|
| trnL2 | 2671 | 2745 | + | 75 | 1 | | |
| ND1 | 2747 | 3703 | + | 957 | -2 | GTG | TAG |
| trnI | 3702 | 3770 | + | 69 | -3 | | |
| trnQ | 3768 | 3839 | - | 72 | -1 | | |
| trnM | 3839 | 3907 | + | 69 | 0 | | |
| ND2 | 3908 | 4951 | + | 1044 | -2 | ATA | TAG |
| trnW | 4950 | 5018 | + | 69 | 4 | | |
| trnA | 5023 | 5091 | - | 69 | 0 | | |
| trnN | 5092 | 5165 | - | 74 | 2 | | |
| trnC | 5197 | 5262 | - | 66 | 0 | | |
| trnY | 5263 | 5326 | - | 64 | 1 | | |
| COX1 | 5328 | 6872 | + | 1545 | -3 | ATG | TAA |
| trnS2 | 6870 | 6938 | - | 69 | 3 | | |
| trnD | 6942 | 7010 | + | 69 | 2 | | |
| COX2 | 7013 | 7696 | + | 684 | 2 | ATG | T(AA) |
| trnK | 7699 | 7763 | + | 65 | 1 | | |
| atp8 | 7765 | 7968 | + | 204 | -43 | ATG | TAA |
| atp6 | 7926 | 8606 | + | 681 | -1 | ATG | TAA |
| COX3 | 8606 | 9390 | + | 785 | -1 | ATG | TTA |
| trnG | 9390 | 9456 | + | 67 | 0 | | |
| ND3 | 9457 | 9804 | + | 348 | 0 | ATA | T(AA) |
| trnR | 9805 | 9873 | + | 69 | 0 | | |
| ND4L | 9874 | 10170 | + | 297 | -7 | ATG | TAA |
| ND4 | 10164 | 11541 | + | 1383 | 0 | ATG | ACT |
| trnH | 11542 | 11609 | + | 68 | 0 | | |
| trnS1 | 11610 | 11668 | + | 59 | 0 | | |
| trnL1 | 11669 | 11739 | + | 71 | 0 | | |
| ND5 | 11740 | 13551 | + | 1812 | 5 | ATA | TAA |
| ND6 | 13557 | 14078 | - | 522 | 1 | CTA | CAT |
| trnE | 14080 | 14148 | - | 69 | 5 | | |
| Cytb | 14154 | 15293 | + | 1140 | 2 | ATG | AGA |
| trnT | 15296 | 15365 | + | 70 | 1 | | |
| trnP | 15367 | 15433 | - | 67 | 244 | | |

Table S11 Mitogenomic organization of *T. cinereus* (Z201312257)

| | Position | | Strand | Length | Intergenic | Codons | |
|---|---|---|---|---|---|---|---|
| | From | To | | | nucleotides | Start | Stop |
| trnF | 1 | 69 | + | 69 | 2 | | |
| rrnS | 72 | 1026 | + | 955 | -1 | | |
| trnV | 1026 | 1092 | + | 67 | 0 | | |
| rrnL | 1093 | 2662 | + | 1570 | 0 | | |
| trnL2 | 2663 | 2737 | + | 75 | 1 | | |
| ND1 | 2739 | 3695 | + | 957 | -2 | GTG | TAG |
| trnI | 3694 | 3762 | + | 69 | -3 | | |

| | | | | | | | |
|---|---|---|---|---|---|---|---|
| trnQ | 3760 | 3831 | - | 72 | -1 | | |
| trnM | 3831 | 3900 | + | 70 | 0 | | |
| ND2 | 3901 | 4944 | + | 1044 | -2 | ATA | TAG |
| trnW | 4943 | 5010 | + | 68 | 4 | | |
| trnA | 5015 | 5083 | - | 69 | 0 | | |
| trnN | 5084 | 5157 | - | 74 | 2 | | |
| trnC | 5190 | 5255 | - | 66 | 0 | | |
| trnY | 5256 | 5319 | - | 64 | 1 | | |
| COX1 | 5321 | 6865 | + | 1545 | -3 | ATG | TAA |
| trnS2 | 6863 | 6931 | - | 69 | 3 | | |
| trnD | 6935 | 7003 | + | 69 | 1 | | |
| COX2 | 7005 | 7688 | + | 684 | 1 | ATG | TAA |
| trnK | 7690 | 7754 | + | 65 | 1 | | |
| ATP8 | 7756 | 7959 | + | 204 | -43 | ATG | TAA |
| ATP6 | 7917 | 8597 | + | 681 | -1 | ATG | TAA |
| COX3 | 8597 | 9381 | + | 785 | -1 | ATG | CTA |
| trnG | 9381 | 9449 | + | 69 | 0 | | |
| ND3 | 9450 | 9797 | + | 348 | 0 | ATT | TAA |
| trnR | 9798 | 9866 | + | 69 | 0 | | |
| ND4L | 9867 | 10163 | + | 297 | -7 | ATG | TAA |
| ND4 | 10157 | 11534 | + | 1383 | 0 | ATG | ACT |
| trnH | 11535 | 11602 | + | 68 | 0 | | |
| trnS1 | 11603 | 11661 | + | 59 | 0 | | |
| trnL1 | 11662 | 11731 | + | 70 | 0 | | |
| ND5 | 11732 | 13543 | + | 1821 | 1 | ATA | TAA |
| ND6 | 13545 | 14069 | - | 525 | 4 | CCT | CAT |
| trnE | 14074 | 14142 | - | 69 | 5 | | |
| Cytb | 14148 | 15287 | + | 1140 | 2 | ATG | AGA |
| trnT | 15290 | 15359 | + | 70 | 1 | | |
| trnP | 15361 | 15427 | - | 67 | 325 | | |

Table S12 Mitogenomic organization of *T.* sp. 2 (1503001)

| | Position | | Strand | Length | Intergenic | Codons | |
|---|---|---|---|---|---|---|---|
| | From | To | | | nucleotides | Start | Stop |
| trnF | 1 | 69 | + | 69 | 2 | | |
| rrnS | 72 | 1027 | + | 956 | -1 | | |
| trnV | 1027 | 1093 | + | 67 | 0 | | |
| rrnL | 1094 | 2665 | + | 1572 | 0 | | |
| trnL2 | 2666 | 2740 | + | 75 | 1 | | |
| ND1 | 2742 | 3698 | + | 957 | -2 | GTG | TAG |
| trnI | 3697 | 3765 | + | 69 | -3 | | |
| trnQ | 3763 | 3834 | - | 72 | -1 | | |
| trnM | 3834 | 3903 | + | 70 | 0 | | |

| | | | | | | | |
|---|---|---|---|---|---|---|---|
| ND2 | 3904 | 4947 | + | 1044 | -2 | ATA | TAG |
| trnW | 4946 | 5013 | + | 68 | 4 | | |
| trnA | 5018 | 5086 | - | 69 | 0 | | |
| trnN | 5087 | 5160 | - | 74 | 2 | | |
| trnC | 5192 | 5258 | - | 67 | 0 | | |
| trnY | 5259 | 5322 | - | 64 | 1 | | |
| COX1 | 5324 | 6868 | + | 1545 | -3 | ATG | TAA |
| trnS2 | 6866 | 6934 | - | 69 | 3 | | |
| trnD | 6938 | 7006 | + | 69 | 1 | | |
| COX2 | 7008 | 7691 | + | 684 | 2 | ATG | TAA |
| trnK | 7694 | 7758 | + | 65 | 1 | | |
| atp8 | 7760 | 7963 | + | 204 | -43 | ATG | TAA |
| atp6 | 7921 | 8601 | + | 681 | -1 | ATG | TAA |
| COX3 | 8601 | 9385 | + | 785 | -1 | ATG | TTA |
| trnG | 9385 | 9452 | + | 68 | 0 | | |
| ND3 | 9453 | 9800 | + | 348 | 0 | ATC | TAA |
| trnR | 9801 | 9869 | + | 69 | 0 | | |
| ND4L | 9870 | 10166 | + | 297 | -7 | ATG | TAA |
| ND4 | 10160 | 11537 | + | 1383 | 0 | ATG | ACT |
| trnH | 11538 | 11605 | + | 68 | 0 | | |
| trnS1 | 11606 | 11664 | + | 59 | 0 | | |
| trnL1 | 11665 | 11734 | + | 70 | 0 | | |
| ND5 | 11735 | 13546 | + | 1812 | 5 | ATT | TAG |
| ND6 | 13552 | 14073 | - | 522 | 1 | CTA | CAT |
| trnE | 14075 | 14145 | - | 71 | 5 | | |
| Cytb | 14151 | 15290 | + | 1140 | 2 | ATG | AGA |
| trnT | 15293 | 15363 | + | 71 | 1 | | |
| trnP | 15365 | 15431 | - | 67 | 319 | | |

Table S13. Partitioning schemes and evolutionary models for Mitochondrial

| Subset partitions | Sites | Best model |
|---|---|---|
| P1: (COX1_codon1, COX2_codon1, CYTB_codon1, COX3_codon1) | 1377 | SYM+G |
| P2: (COX1_codon2, COX3_codon2, CYTB_codon2, COX2_codon2) | 1377 | TRN+G |
| P3: (ATP8_codon3, ATP6_codon3, COX3_codon3, COX1_codon3, COX2_codon3) | 1291 | TRN+G |
| P4: (ATP8_codon2, ATP8_codon1, ND4L_codon1, ND1_codon1, ND3_codon1, ND5_codon1, ND4_codon1, ATP6_codon1) | 1953 | GTR+G |
| P5: (ND4L_codon2, ND1_codon2, ATP6_codon2, ND3_codon2, ND5_codon2, ND4_codon2) | 1819 | GTR+G |
| P6: (ND3_codon3, ND5_codon3, ND4_codon3, CYTB_codon3, ND1_codon3, ND4L_codon3) | 1972 | GTR+G |
| P7: (ND2_codon1) | 318 | TRN+G |
| P8: (ND2_codon2) | 318 | HKY |
| P9: (ND2_codon3) | 318 | HKY+G |

| | | |
|---|---|---|
| P10: (ND6_codon2, ND6_codon1) | 344 | HKY+G |
| P11: (ND6_codon3) | 172 | HKY+G |
| P12: (12s_codon2, 12s_codon1, 12s_codon3) | 948 | HKY |
| P13: (16s_codon2, 16s_codon1, 16s_codon3) | 1569 | TRN+G |

Table S14. Comparison of the fit of different models of biogeographical range evolution and model specific estimates for different parameters (d = dispersal, e = extinction, j = weight of jump dispersal (founder speciation)).

| | LnL | numparams | d | e | j | AICc | AICc_wt |
|---|---|---|---|---|---|---|---|
| DEC | -7.961397459 | 2 | 0.281384636 | 3.221411058 | 0 | 22.92279492 | 0.223200088 |
| DEC+J | -6.72780632 | 3 | 1.00E-12 | 3.481739962 | 0.183493744 | 27.45561264 | 0.023142246 |
| DIVALIKE | -7.329972387 | 2 | 0.0404745 | 4.998658648 | 0 | 21.65994477 | 0.419680496 |
| DIVALIKE+J | -6.545880699 | 3 | 1.00E-12 | 4.994631849 | 0.160518189 | 27.0917614 | 0.027759702 |
| BAYAREALIKE | -7.721848345 | 2 | 0.200625971 | 3.099252077 | 0 | 22.44369669 | 0.283615015 |
| BAYAREALIKE+J | -6.75140761 | 3 | 1.00E-07 | 3.305134263 | 0.177996784 | 27.50281522 | 0.022602454 |

Table S15. Comparison of the length of the D-loop region.

| Species | Length (bp) |
|---|---|
| *T. chapensis* | 1935 |
| *T. cinereus* | 1062 |
| *T. daloushanensis* | 1005 |
| *T. nanus* | 1220 |
| *T. huangshanensis* | 1064 |
| *T. fengjiensis* | 1054 |
| *T. sp. 2* | 1059 |

Table S16. Compare the ND5 in the mitochondrial genomes of various species.

| Species | Length (bp) | A+T (%) | C+G(%) | Start | Stop |
|---|---|---|---|---|---|
| *T. chapensis* | 1812 | 60.1 | 39.9 | ATT | TAA |
| *T. cinereus* | 1812 | 63.6 | 36.4 | ATA | TAA |

| | | | | | |
|---|---|---|---|---|---|
| *T. daloushanensis* | 1812 | 63.6 | 36.4 | ATT | TAA |
| *T. nanus* | 1812 | 62.6 | 37.4 | ATA | TAG |
| *T. huangshanensis* | 1812 | 65.2 | 34.8 | ATT | TAA |
| *T. fengjiensis* | 1812 | 64.1 | 35.9 | ATA | TAA |
| *T. sp. 2* | 1812 | 64.6 | 35.4 | ATT | TAG |
